# Multiplex screening of 275 plasma protein biomarkers to identify a signature for early detection of colorectal cancer

**DOI:** 10.1002/1878-0261.12591

**Published:** 2019-11-13

**Authors:** Megha Bhardwaj, Korbinian Weigl, Kaja Tikk, Axel Benner, Petra Schrotz‐King, Hermann Brenner

**Affiliations:** ^1^ Division of Preventive Oncology German Cancer Research Center (DKFZ) and National Center for Tumor Diseases (NCT) Heidelberg Germany; ^2^ Medical Faculty Heidelberg University of Heidelberg Germany; ^3^ Division of Clinical Epidemiology and Aging Research German Cancer Research Center (DKFZ) Heidelberg Germany; ^4^ German Cancer Consortium (DKTK) German Cancer Research Center (DKFZ) Heidelberg Germany; ^5^ Division of Biostatistics German Cancer Research Center (DKFZ) Heidelberg Germany

**Keywords:** colorectal cancer, diagnostic biomarkers, early detection, proximity extension assay, screening, sensitivity and specificity

## Abstract

Blood‐based protein biomarkers may be an attractive option for early detection of colorectal cancer (CRC). Here, we used a two‐stage design to measure 275 protein markers by proximity extension assay (PEA), first in plasma samples of a discovery set consisting of 98 newly diagnosed CRC cases and 100 age‐ and gender‐matched controls free of neoplasm at screening colonoscopy. An algorithm predicting the presence of early‐ or late‐stage CRC was derived by least absolute shrinkage and selection operator regression with .632+ bootstrap method, and the algorithms were then validated using PEA again in an independent validation set consisting of participants of screening colonoscopy with and without CRC (*n* = 56 and 102, respectively). Three different signatures for all‐, early‐, and late‐stage CRC consisting of 9, 12, and 11 protein markers were obtained in the discovery set with areas under the curves (AUCs) after .632 + bootstrap adjustment of 0.92, 0.91, and 0.96, respectively. External validation among participants of screening colonoscopy yielded AUCs of 0.76 [95% confidence interval (95% CI), 0.67–0.84], 0.75 (95% CI, 0.62–0.87), and 0.80 (95% CI, 0.68–0.89) for all‐, early‐, and late‐stage CRC, respectively. Although the identified protein markers are not competitive with the best available stool tests, these proteins may contribute to the development of powerful blood‐based tests for CRC early detection in the future.

## Graphical abstract

A signature consisting of 12 plasma protein biomarkers derived in a clinical setting and independently validated in a true screening setting (participants of screening colonoscopy) yielded area under the curves of 0.91 and 0.75 for distinguishing early‐stage colorectal cases from controls free of neoplasms. These 12 protein markers may contribute to the development of blood‐based tests for population‐based screening.

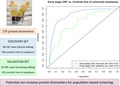

**Abbreviations**
AA: advanced adenomas
AREG: amphiregulin
ASTER: Mit ASS Darmtumore früher erkennen
AUC: area under the receiver operating curve
AUC^BS^: .632+ bootstrap‐adjusted AUC
BLITZ: Begleitende Evaluierung innovativer Testverfahren zur Darmkrebs‐Früherkennung
CEA: carcinoembryonic antigen
CRC: colorectal cancer
CV: coefficient of variance
GZMB: granzyme B
iDa: Durch innovative Testverfahren Darmkrebs früher erkennen
IL6: interleukin‐6
ITGA11: integrin alpha 11
ITGAV: integrin alpha V
KRT19: keratin, type I cytoskeletal 19
LASSO: least absolute shrinkage and selection operator
MASP1: mannan‐binding lectin serine protease 1
MCP1: monocyte chemotactic protein 1
NPX: normalized protein expression
NTproBNP: N‐terminal prohormone brain natriuretic peptide
OPN: osteopontin
PEA: proximity extension assay
PON3: paraoxonase 3
QCC: quality control criteria
RARRES2: retinoic acid receptor responder protein 2
S100A4: protein S100‐A4
TR: transferrin receptor protein 1
TRAIL: TNF‐related apoptosis‐inducing ligand
TRAP: tartrate‐resistant acid phosphatase type 5

## 1 Introduction

With 1.85 million incident cases and ~ 880 000 deaths per year, colorectal cancer (CRC) is the third most common cancer and second leading cause of cancer mortality globally (Bray *et al.*, 2018). Randomized trials and observational studies have established the potential of screening with stool‐based tests or endoscopy in reducing CRC incidence and mortality (Atkin *et al.*, 2017; Brenner and Chen, 2018; Brenner *et al.*, 2014; Shaukat *et al.*, 2013; Zauber *et al.*, 2012), and stool tests or endoscopy‐based CRC screening is offered in an increasing number of countries (Klabunde *et al.*, 2015; Schreuders *et al.*, 2015). However, the participation rates in screening programs are often low because of limitations such as invasiveness, costs and resources, inconvenience, and adherence (Bretthauer *et al.*, 2016; Klabunde *et al.*, 2015; Navarro *et al.*, 2017; Pox *et al.*, 2012; Schreuders *et al.*, 2015; Segnan *et al.*, 2007).

Minimally invasive blood‐based tests might improve the participation rates in population‐based screening programs, given their straightforward applicability in routine medical assessments. Extensive research has been conducted to identify blood‐based biomarkers for early detection of CRC. In particular, numerous studies searched for blood‐based protein biomarkers or biomarker signatures, and some of them reported seemingly good diagnostic performance (Bhardwaj *et al.*, 2017a; Bhardwaj *et al.*, 2017b). However, the majority of studies were conducted in clinical settings and lacked proper validation in true screening settings, which may have resulted in highly overoptimistic results of diagnostic performance. Furthermore, few studies reported on sensitivity by CRC stage.

Among the very few studies that performed both internal validation and external validation, two studies from our group in which 92 tumor‐associated proteins were screened simultaneously found promising results (Chen *et al.*, 2017; Chen *et al.*, 2015). However, compared with the current study both of the previous studies had smaller numbers of CRC cases in the screening cohort and no stage‐specific prediction algorithms were reported. Therefore, we prospectively evaluated a broader spectrum of 275 proteins with the objective to identify multimarker signature with optimal diagnostic performance for early detection of CRC in a discovery set. The estimates were independently validated in prospectively selected samples (from *N* = 9245) exclusively recruited in a true screening setting.

## 2 Materials and methods

### 2.1 Study design

The protein marker signature was developed in a two‐step approach, with construction of multimarker algorithms in a discovery set and evaluation and validation of findings in an independent validation set. The discovery set included CRC patients recruited in a clinical setting and compared them with participants found to be free of colorectal neoplasms at screening colonoscopy. The validation set was exclusively based on participants of screening colonoscopy in order to evaluate diagnostic performance in a true screening setting.

### 2.2 Study population: discovery set

The discovery set consisted of 98 CRC cases recruited prior to any therapeutic measures from the Durch innovative Testverfahren Darmkrebs früher erkennen (iDa) study in hospitals in southwestern Germany between 2013 and 2016. As controls, 100 participants of screening colonoscopy who were found to be free of neoplasms were selected using frequency matching by age and sex from the ‘Mit ASS Darmtumore früher erkennen’ (ASTER) study. The ASTER study, a multicenter prospective randomized controlled trial (EudraCT No. 2011‐005603‐32), recruited participants aged 40–80 with a planned screening (75%) or diagnostic (25%) colonoscopy from gastrointestinal practices in Germany from 2013 to 2016 to determine whether the application of a single 300 mg dose of aspirin before performing a fecal immunochemical tests (FIT) could improve FIT sensitivity for detecting advanced neoplasms (Tikk *et al.*, 2018). For both iDa and ASTER, the use of samples for the evaluation of early detection markers for CRC has been approved by the ethics committees of the Medical Faculty Heidelberg (S‐489/2012 and AFmu‐271/2012, for iDa and ASTER, respectively) and from the responsible state medical boards. Both the studies were conducted adhering to the standards set by Declaration of Helsinki and were undertaken with the understanding and written consent of each subject. The complete Standards for the Reporting of Diagnostic Accuracy Studies (STARD) diagrams displaying the selection of study participants in iDa and ASTER studies are shown in Figs S1 and S2.

### 2.3 Study population: validation set

**Figure 1:**
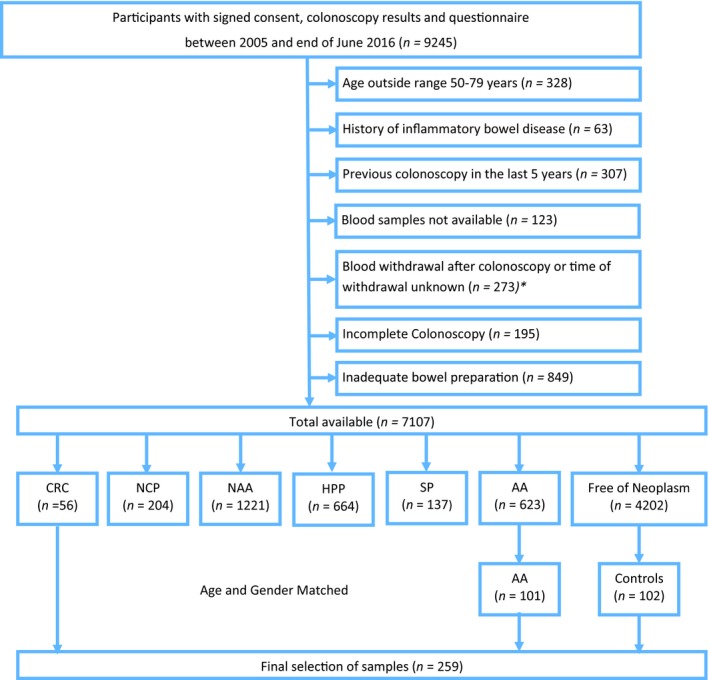
STARD flow diagram BLITZ study. HPP, hyperplastic polyps; NAA, nonadvanced adenoma; NCP, nonclassified polyp; SP, serrated poly. *The exclusion criteria  for selection of CRC cases were not applicable after this point.

For the independent external validation of potentially promising signatures, blood samples were selected from participants of screening colonoscopy collected in the Begleitende Evaluierung innovativer Testverfahren zur Darmkrebs‐Früherkennung (BLITZ) study. Details of the BLITZ study design have been reported previously (Brenner *et al.*, 2010b; Chen *et al.*, 2017; Chen *et al.*, 2015; Gies *et al.*, 2018; Hundt *et al.*, 2009). In brief, BLITZ is an ongoing screening study of participants of the German screening colonoscopy program that is offered to men and women aged 55 and older. Participants are recruited in 20 gastroenterology practices since end of the year 2005. By the end of June 2016, 9245 participants were recruited into the BLITZ study, and after the application of exclusion criteria (specified in Fig. 1), CRC and advanced adenomas (AA), the precursors of CRC had been detected in 56 and 623 participants, respectively. In the present study, the validation of protein signatures derived in the discovery set was carried out in blood samples from 56 participants with CRC and 102 controls who were free of colorectal neoplasm. Controls were frequency‐matched to the CRC cases by sex and age. In addition, we also selected 101 participants with AA (defined as adenoma with > 1 cm in diameter, tubulovillous or villous components, or high‐grade dysplasia (Brenner *et al.*, 2010a)) who were likewise frequency‐matched to the CRC cases by sex and age. The BLITZ study and use of its samples for the evaluation of early detection markers for CRC have been approved by the ethics committees of the Medical Faculty Heidelberg (S‐178/2005), and of the physicians’ boards of Baden‐Wuerttemberg (M118‐05‐f), Rhineland‐Palatinate [837.047.06(5145)], Hessen (MC 254/2007), and Saarland (217/13). The BLITZ study adheres to the standards set by Declaration of Helsinki, and all the study participants have voluntarily given their written informed consent. The STARD diagram showing selection of study participants from the BLITZ study is presented in Fig. 1.

### 2.4 Sample collection and storage

The blood draw was performed at first diagnosis of CRC before any treatment for cancer in iDa and before colonoscopy in ASTER and BLITZ. After blood draw, EDTA plasma samples were transported to the laboratory while preserved in cold chain, followed by centrifugation at 2000–2500 ***g*** for 10 min, and were then stored at – 80 °C until the protein measurements. The laboratory staff was blind to any information regarding the study population.

### 2.5 Laboratory assay

Protein concentrations in plasma samples were measured utilizing the proximity extension assays (PEAs) offered by Olink. Olink’s multiplex panels allow simultaneous analysis of 92 biomarkers in 1 µl samples, and the full protocol of the PEA has been reported previously (Assarsson *et al.*, 2014). Briefly, the 96 pairs of oligonucleotide‐labeled antibodies (92 biomarkers and four internal controls) are allowed to pairwise bind to target proteins and when in close proximity a PCR reporter sequence is formed due to DNA polymerization, which is quantified by real‐time PCR. For our study, we used the Olink multiplex panels ‘Oncology II’, ‘Immune response’, and ‘Cardiovascular III’ resulting in a total of 276 proteins analyzed. The full list of markers from each panel is provided in Table S1. Laboratory analyses were performed blinded with respect to disease status or findings at colonoscopy in the laboratory of the manufacturer of the panels. Each assay form these panels has been validated, and information on characteristics such as detection limits, dynamic range, repeatability, and reproducibility is available from the manufacturer’s website (https://www.olink.com/data-you-can-trust/validation/).

### 2.6 Statistical analysis

All linear protein values were log‐transformed to produce normalized protein expression (NPX). In the current study, the NPX values of each individual protein were compared between CRC and controls in the discovery set using Wilcoxon rank‐sum test with adjustment for multiple testing by the Benjamini and Hochberg method (Benjamini and Hochberg, 1995). For each individual protein biomarker, a logistic regression model was used to construct the prediction algorithm and .632+ bootstrap was applied to adjust for potential overestimation of diagnostic performance (Efron and Tibshirani, 1997). Areas under the receiver operating characteristic curves (AUCs) and their 95% confidence intervals (95% CI) and sensitivity (true positive rate) of each individual biomarker at cutoffs yielding 80% and 90% specificities (true‐negative rate) were calculated.

In order to derive multimarker algorithms for the prediction of the presence of CRC, least absolute shrinkage and selection operator (LASSO) logistic regression models were applied to markers that remained significant after multiple testing in the discovery set. The LASSO regression was adapted in order to obtain models with the best prediction accuracy and was combined with .632+ bootstrap (Efron and Tibshirani, 1997) to adjust for overfitting. Three prediction algorithms were derived, one each for all (I–IV)‐, early (I–II)‐, and late (III–IV)‐stage CRC vs controls, respectively. For evaluating the diagnostic performance of each algorithm, the sensitivity, specificity, and apparent AUC, that is, the AUC not adjusted for overfitting (AUC*) with 95% CI, as well as .632+ bootstrap‐adjusted AUC (AUC^BS^), were calculated. In order to externally validate the prediction algorithms in a real‐life screening setting, their performance was finally evaluated in the validation set that exclusively included participants of screening colonoscopy. The performance of CRC early‐stage algorithm was additionally evaluated for AA participants in the validation set.

In the validation set, diagnostic performance of the proteins and protein algorithms was evaluated separately for CRC and for AA, in each case in comparison with controls free of neoplasms. According to common practice in biomarker research, this comparison was done using controls that were matched to the CRC cases with respect to for age and sex distribution in the first place. However, the age and sex distribution of participants free of colorectal neoplasms actually differs from the corresponding distributions of CRC cases in a true screening setting. With the objective of providing valid estimates of the algorithms’ performance in a true screening setting, the observations from participants with AA and controls free of neoplasms were therefore weighted in the validation set of the current study in such a way that their distribution reflects the sex and age distribution of all participants with AA and controls free of neoplasms among the participants of screening colonoscopy, respectively (Brenner *et al.*, 2013). All statistical analyses were performed with statistical software r language and environment (version 3.5.0, R core team) (R Core Team, 2016). For all tests, *P*‐values of 0.05 or less were considered to be statistically significant.

## 3 Results

### 3.1 Characteristics of the study populations

Figures S1 and S2 and Fig. 1 provide the STARD diagrams displaying the selection of study participants enrolled in iDa, ASTER, and BLITZ, respectively. The discovery set consisted of 98 and 100 clinically recruited CRC cases from iDa and controls from ASTER, respectively. The validation set included 56 and 101 participants of screening colonoscopy with CRC and AA, respectively, and 102 controls free of neoplasms from the BLITZ study. The characteristics of populations from both the discovery and the validation sets are shown in Table 1. The distribution of characteristics was largely similar across both sets with the median age being around 65 years and males representing ≥ 60% of population in both sets. Table 1 additionally includes the age and sex distribution of AA cases and controls in the entire screening population from which the matched AA cases and controls were drawn

**Table 1:** Characteristics of the study population in both sets. Ca, cancer; *N*, number; SD, standard deviation.

Group	Discovery set		Validation set			Participants of screening colonoscopy	
	iDa (Clinical) CRC	ASTER (Mostly screening) controls	BLITZ matched set (Screening)			BLITZ (Screening)	
			CRC	AA	Controls	AA	Controls
Total	*N* (%)	*N* (%)	*N* (%)	*N* (%)	*N* (%)	*N* (%)	*N* (%)
	98	100	56	101	102	623	4202
Age in years							
50–59	23 (23)	29 (29)	10 (18)	22 (21)	21 (21)	237 (38)	1916 (46)
60–69	42 (43)	45 (45)	28 (50)	49 (49)	50 (49)	247 (40)	1614 (38)
70–79	33 (34)	26 (26)	18 (32)	30 (30)	31 (30)	139 (22)	672 (16)
Mean	64.6	64.0	66.0	65.5	65.4	63.3	61.9
Median	64.5	65.5	65.0	65.0	65.5	62.0	60.0
SD	6.9	7.5	5.8	6.6	6.9	5.9	6.5
Gender distribution							
Male	60 (60)	60 (60)	36 (64)	65 (64)	66 (65)	393 (63)	1808 (43)
Female	40 (40)	40 (40)	20 (36)	36 (36)	36 (35)	230 (37)	2394 (57)
Stage distribution							
Stage I	17 (17)	–	17 (30)	–	–	–	–
Stage II	32 (33)	–	6 (11)	–	–	–	–
Stage III	23 (23)	–	26 (46)	–	–	–	–
Stage IV	26 (27)	–	7 (13)	–	–	–	–
Early stage (I/II)	49 (50)	–	23 (41)	–	–	–	–
Late stage (III/IV)	49 (50)	–	33 (59)	–	–	–	–
Cancer site							
Proximal colon	41 (42)	–	9 (16)	–	–	–	–
Distal colon	23 (23)	–	24 (43)	–	–	–	–
Rectum	33 (34)	–	21 (38)	–	–	–	–
Unknown	1 (1)	–	2 (4)	–	–	–	–

### 3.2 Assay performance

The quality control criteria (QCC) for both the datasets were considered good with 95–100% of the samples meeting QCC across the three Proseek panels and 82–100% of proteins being above the limit of detection. For the three multiplex panels, the average intra‐assay coefficient of variance (CV) was 5% and 7% and the average interassay CV was 19% and 15% for discovery and validation sets, respectively. Due to redundancy between panels and one analyte not meeting QCC, reportable results were obtained for 275 proteins. Amphiregulin (AREG) and interleukin‐6 (IL6) were the two proteins measured on multiple panels, and both showed good concordance (Pearson’s product–moment correlation, *r* = 0.99 and 0.98, respectively). The complete experimental workflow of the study has been described in Fig. S3.

### 3.3 Individual marker analysis for all‐stage CRC

For the discovery set, analysis was conducted with 97 CRC cases and 99 controls because two participants had to be excluded on account of several missing protein measurements. In the validation set, the analysis was based on 56 CRC, 101 AA, and 102 controls. Results of the univariate analysis comparing the expression difference in each marker in plasma between cases and controls in both sets are summarized in Table S2. When results for the 275 markers were adjusted for multiple testing, overall 83 markers showed statistically significant differences in expression levels in the discovery set (adjusted *P*‐values ≤ 0.05 in Table S2). After correction for overoptimism by .632+ bootstrap, AUC^BS^ of these 83 markers ranged from 0.80 [0.73–0.89] to 0.58 [0.48–0.70] with sensitivities at cutoffs yielding 80% specificity ranging from 68% to 24%. However, in the validation set only nine of these 83 markers were successfully replicated with adjusted *P*‐values ≤ 0.05.

### 3.4 Multimarker predictor algorithms from the discovery set

**Figure 2:**
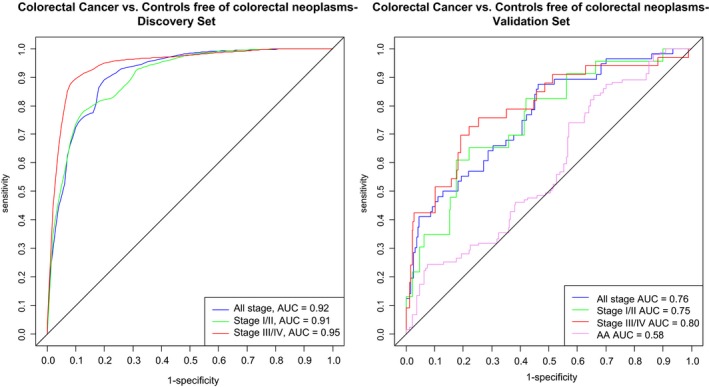
Comparison of the receiver operating characteristic curves for detecting (all/early/late) stage CRC and AA vs free of neoplasm controls for discovery and validation set with 9‐, 12‐, and 11‐marker signatures.

The Lasso logistic regression model was applied on all 83 protein markers with adjusted *P*‐values ≤ 0.05 from the discovery set, in order to construct a multimarker prediction algorithm for comparing all‐, early‐, and late‐stage CRC to controls. Table 2 and Fig. 2 show that the best performances were obtained for 9‐, 12‐, and 11‐marker combinations. The 9‐marker algorithm for all stages selected the following proteins: AREG, carcinoembryonic antigen (CEA), granzyme B (GZMB), integrin alpha V (ITGAV), keratin, type I cytoskeletal 19 (KRT19), monocyte chemotactic protein 1 (MCP1), osteopontin (OPN), paraoxonase 3 (PON3), and transferrin receptor protein 1 (TR). The AUC^BS^ was 0.92, and diagnostic sensitivities of 89% and 73% were obtained at cutoffs yielding 90% and 80% specificity, respectively.

**Table 2:** Diagnostic performance of multimarker signatures for detecting CRC (all stages/early/late/AA) in discovery and validation sets. Se, sensitivity; Sp, specificity.

Stage‐specific CRC vs controls free of neoplasms	Protein markers discovered in the algorithm/signatures	Discovery set				Validation set (as in matched samples)			Validation set (as in screening population)			
		AUC^BS^	AUC^*^	Se % at 80% Sp	Se % at 90% Sp	AUC (95% CI)	Se % at 80% Sp	Se % at 90% Sp	AUC (95% CI)	Se % at 80% Sp	Se % at 90% Sp
All‐stage CRC	AREG+ CEA+ GZMB+ ITGAV+ KRT19+ MCP1+ OPN+ PON3+ TR	0.92	0.92	89	73	0.75 (0.67–0.82)	50	41	0.76 (0.67–0.84)	55	45
Early‐stage CRC	AREG+ CEA+ GZMB+ ITGAV+ KRT19+ MASP1+ MCP1+ PON3+ RARRES2+ S100A4+ TR+ TRAP	0.91	0.91	82	74	0.73 (0.62–0.83)	61	35	0.75 (0.62–0.87)	61	35
Late‐stage CRC	AREG+ CEA+ IL6+ ITGA11+ ITGAV+ KRT19+ MCP1+ NTproBNP+ OPN+ TR+ TRAIL	0.95	0.96	95	89	0.81 (0.72–0.89)	71	51	0.80 (0.68–0.89)	70	51
AA	AREG+ CEA+ GZMB+ ITGAV+ KRT19+ MASP1+ MCP1+ PON3+ RARRES2+ S100A4+ TR+ TRAP	–	–	–	–	0.45 (0.37–0.53)	13	5	0.58 (0.47–0.68)	28	24

For early‐stage CRC, as shown in Table 2 the algorithm comprised 12 proteins, 8 of which were identical with algorithm from all stages, that is, AREG, CEA, GZMB, ITGAV, KRT19, MCP1, PON3, TR, and additionally mannan‐binding lectin serine protease 1 (MASP1), retinoic acid receptor responder protein 2 (RARRES2), protein S100‐A4 (S100A4), and tartrate‐resistant acid phosphatase type 5 (TRAP), respectively. The AUC^BS^ for the 12‐marker algorithm for the comparison of early‐stage CRC cases vs controls was 0.91, and when defining cutoffs to 80% and 90% specificities, sensitivities of 82% and 74% were observed. For late stages, the AUC^BS^ was 0.95 for an algorithm with 11 markers, namely AREG, CEA, ITGAV, KRT19, MCP1, OPN, TR, IL6, integrin alpha 11 (ITGA11), N‐terminal prohormone brain natriuretic peptide (NTproBNP), and TNF‐related apoptosis‐inducing ligand (TRAIL). The sensitivities for the 11‐marker algorithm for the comparison of late‐stage CRC cases vs controls were 95% and 89% at cutoffs yielding 80% and 90% specificity, respectively.

### 3.5 Diagnostic performance of multimarker signatures in the validation set

For external validation, the performance was evaluated in an independent population consisting of participants from a true screening study. The prediction algorithms derived from discovery set are presented in Table S3. Upon application of estimates from the predictor algorithms obtained from discovery set, the AUCs as shown in Table 2 and Fig. 2 for 9‐, 12‐, and 11‐marker signatures were 0.76 (95% CI, 0.67–0.84), 0.75 (95% CI, 0.62–0.87), and 0.80 (95% CI, 0.68–0.89). At cutoff yielding 80% specificity, sensitivities of 55%, 61%, and 70% and at defining cutoff at 90% specificity, sensitivities of 45%, 35%, and 51% were observed for comparing all‐, early‐, and late‐stage CRC cases vs controls, respectively. When the predictor algorithm for early stage was applied for comparing participants with AA to controls, an AUC of 0.58 (95% CI, 0.47–0.68) was observed.

### 3.6 Site‐specific diagnostic performance of markers in both sets

In order to observe the site‐specific diagnostic performances, further stratification was performed with respect to the location of cancer, that is, proximal colon, distal colon, or rectum, respectively. The results of the site‐specific analyses in the discovery and validation sets for individual markers and multimarker signature are presented in Tables S4 and S5, respectively. As shown in Table S4, when further stratification with respect to location was performed and all 275 markers were adjusted for multiple testing, overall 47, 30, and 25 protein markers showed statistically significant differences in expression levels for the proximal colon, distal colon, and rectum, respectively. In the validation set due to comparatively lower number of cases, only 1, 2, and 6 of these markers for the proximal colon, distal colon, and rectum, respectively, could be replicated. The site‐specific AUCs of the 9‐marker signature for the detection of proximal, distal, and rectal CRCs were 0.98, 0.95, and 0.95 in the discovery set and 0.70, 0.77, and 0.78 in the validation set, respectively (Table S5).

### 3.7 Stage‐specific diagnostic performance of identified markers in both sets

The individual marker performances of the 17 markers identified in all three prediction algorithms for early‐ and late‐stage CRC detection in discovery and validation sets are presented in Table 4. In the discovery set, diagnostic performance of 13 out of 17 markers was comparatively higher for late‐stage CRC detection and usually the performances were better in discovery than validation sets for both early‐stage CRC detection and late‐stage CRC detection. For early‐stage CRC, four markers that presented with AUC ≥ 0.7 in the discovery set were AREG, ITGA11, ITGAV, and PON3. However, in the validation set only AREG of these four markers presented with AUC < 0.7 and sensitivity of 55% at cutoff yielding 80% specificity. For late‐stage CRC, the four markers with AUC ≥ 0.7 in both the discovery and validation sets were AREG, CEA, KRT19, and TR. In validation set, the markers CEA (AUC = 0.76), KRT19 (AUC = 0.74), and TR (AUC = 0.74) best detected late‐stage CRC cases with sensitivities of 62%, 52%, and 50% at cutoff yielding 80% specificity.

### 3.8 Function of the protein markers

There were in total 17 proteins found in three different prediction models, and as depicted in Table 3, these proteins have a wide variety of molecular functions with five, four, and three of them being cytokines, hydrolases, and receptors, respectively. As depicted in Fig. S4, when Ingenuity Pathway Analysis (Qiagen Inc., https://www.qiagenbioinformatics.com/products/ingenuity-pathway-analysis, version 01‐10; Ingenuity Systems, Redwood City, CA, USA) was used to understand the interaction between these proteins in established canonical pathways, it was observed that though functionally different these proteins interact at cellular or extracellular level in several different pathways (Fig. S4). However, some proteins such as cytokines IL6 and MCP1 and receptors ITGAV and ITGA11 interact directly in more than one pathway. Additionally, when the role of these proteins in different organ toxicities was looked upon (Fig. S5), it was found that several proteins such as IL6, ITGAV, PON3, OPN, TR, and TRAIL are involved in toxicities such as liver necrosis/proliferation/fibrosis and renal necrosis.

**Table 3:** Distribution of proteins from multimarker signatures across different panels and according to the stages involved and their functions. CVDIII, cardiovascular III panel; ID, identification; IR, immune response panel; ONCO II, oncology II panel.

Name	Uniprot ID	Pea panel	Molecular function	Biological process	All stage	Early stage	Late stage
AREG	http://www.uniprot.org/uniprot/P15514	ONCO II	Cytokine, growth factor	Cell–cell signaling, cell proliferation	x	x	x
CEA	http://www.uniprot.org/uniprot/P06731	ONCO II	GPI anchor binding; glycoprotein	Homotypic cell–cell adhesion	x	x	x
GZMB	http://www.uniprot.org/uniprot/P10144	ONCO II	Hydrolase, protease, serine protease	Apoptosis, cytolysis	x	x	
IL‐6	http://www.uniprot.org/uniprot/P05231	IR	Cytokine, growth factor	Acute‐phase response			x
Integrin alpha‐11	http://www.uniprot.org/uniprot/Q9UKX5	IR	Integrin, receptor	Cell adhesion			x
ITGAV	http://www.uniprot.org/uniprot/P06756	ONCO II	Host cell receptor for virus entry, integrin, receptor	Calcium, metal binding	x	x	x
KRT19	http://www.uniprot.org/uniprot/P08727	IR	Protein complex binding	Host–virus interaction	x	x	x
MASP1	http://www.uniprot.org/uniprot/P48740	IR	Hydrolase, protease, serine protease	Complement activation lectin pathway, immunity, innate immunity		x	
MCP1	http://www.uniprot.org/uniprot/P13500	CVD III	Cytokine/chemokine	Chemotaxis, inflammatory response	x	x	x
NTproBNP	http://www.uniprot.org/uniprot/NA	CVDIII	NA	NA			x
OPN	http://www.uniprot.org/uniprot/P10451	CVD III	Cytokine	Biomineralization, cell adhesion	x		x
PON3	http://www.uniprot.org/uniprot/Q15166	CVD III	Hydrolase	Calcium, metal binding	x	x	
RARRES2	http://www.uniprot.org/uniprot/Q99969	CVD III	Receptor binding	Chemotaxis, differentiation, inflammatory response		x	
S100A4	http://www.uniprot.org/uniprot/P26447	ONCO II	Calcium‐dependent protein binding	Epithelial‐to‐mesenchymal transition		x	
TR	http://www.uniprot.org/uniprot/P02786	CVD III	Host cell receptor for virus entry, receptor	Endocytosis, host–virus interaction	x	x	x
TRAP	http://www.uniprot.org/uniprot/P13686	CVD III	Hydrolase	Iron, metal binding		x	
TRAIL	http://www.uniprot.org/uniprot/P50591	ONCO II	Cytokine	Apoptosis			x

## 4 Discussion

In this study, we performed PEA on two different sets of populations for the identification and validation of protein multimarker signatures for the detection of CRC and its precursors. The study was performed in a two‐stage design, and the independent external validation was performed on 56 CRC and 102 controls recruited among 9245 participants of a true screening study. We identified three different multimarker signatures for stage‐specific CRC. A total of 17 different proteins were identified in different prediction models. A 9‐marker panel with an AUC^BS^ of 0.92 and an AUC 0.76 (95% CI, 0.67–0.84) in the discovery and the validation set, respectively, best detected CRC at all stage. For stage‐specific models, the best diagnostic performance was observed in forms of 12‐marker and 11‐marker signatures with AUC^BS^ of 0.91 and 0.95 in the discovery set and AUC of 0.75 (95% CI, 0.62–0.87) and 0.80 (95% CI, 0.68–0.89) for detecting early‐ and late‐stage CRC in the validation set, respectively.

Previous research pertaining to blood‐based protein markers has been mostly conducted on samples from clinical settings (Bhardwaj *et al.*, 2017a; Bhardwaj *et al.*, 2017b). Only few studies went a step further and performed rigorous independent validation in true screening settings. The cases recruited in clinical settings are mostly symptomatic, have typically undergone several diagnostic procedures, and in several instances they may have undergone initial therapeutic intervention and lifestyle modification prior to sample collection. When such cases are compared to healthy controls, diagnostic performance indicators tend to be higher which might lead to spectrum bias (Kohn *et al.*, 2013; Whiting *et al.*, 2004). Likewise, the controls recruited in clinical settings often consist of patients suffering from some other systemic diseases. Such factors may influence the diagnostic performance indicators and may lead to false‐positive findings (Chen *et al.*, 2016; Tao *et al.*, 2011). Additionally, hospital controls usually have higher response rates than population controls, which might contribute to sampling bias. In our study, we therefore made major efforts to avoid such biases by externally validating the performance of the predictor algorithms in an independent set exclusively of participants recruited in a true screening setting, that is, before screening colonoscopy. The pre‐analytical processing of samples influences the measurements in protein biomarker research (Enroth *et al.*, 2016; Lee *et al.*, 2010; Shen *et al.*, 2018). In the current study although participants in the discovery and validation sets were selected from three different studies, the collection, handling, processing, and storage of the samples across the three studies were performed with similar standardized operating procedure.

The PEAs utilize a pair of oligonucleotide‐labeled antibodies or probes for the detection of each protein. These probes have to be in close proximity, and only this dual recognition of the target protein leads to initiation of an amplified signal detection. PEA quantifies across five logs of abundance with good reproducibility (CV < 20%), and a very low volume of 1 µL is required per sample. The technical assay sensitivity for the PEAs is in the picogram·mL^−1^ range. The dual recognition, requirements of low sample volume, and good assay sensitivity make PEA a very target‐specific and efficient method. Nevertheless, the type and number of proteins included in each of these commercially offered PEA panels are predetermined and cannot be selected. Each panel allows simultaneous detection of only 92 proteins, so further developments are warranted for full proteome coverage. Additionally, even though the reproducibility of PEAs is comparatively better than other antibody‐based methods, replication of our findings using other quantification techniques should be aimed for in future studies.

In two former studies from our research group on protein marker signatures where external validation was conducted (Chen *et al.*, 2015; Surinova *et al.*, 2015), AUCs of 8‐marker and 6‐marker algorithms were 0.76 (Chen *et al.*, 2015) and 0.84 (Surinova *et al.*, 2015), respectively. However, unlike the current study, these studies included CRC cases recruited in clinical settings in the external validation. In another study from our group with a totally distinct discovery set but a partly overlapping external validation set (Chen *et al.*, 2017) recruited in the BLITZ study, an AUC of 0.82 had been found for a five‐marker signature [AREG, growth differentiation factor 15, fas antigen ligand, fms‐related tyrosine kinase 3 ligand, and TP53 autoantibody (antiTP53)]. Even though the current study was based on a comparatively larger sample size and included approximately three times as many proteins (but not antiTP53) in three different panels, the AUC in the validation set was slightly lower. This was the case even if antiTP53 had been left out from our previously identified algorithm. Possibly, the combination of the previously detected and the newly detected proteins may allow for further improvement of diagnostic performance for early detection. Algorithms based on such combination would though have to be addressed in additional independent samples in order to provide unbiased estimates of diagnostic performance.

The diagnostic performance of our 12‐marker algorithm was comparable to that of FITs in the discovery set with 82% sensitivity at 80% specificity for early stage. Previous studies on blood‐based tests that are not exclusively based on proteins, such as COLODETECT (4 proteins + 3 phages) (Barderas *et al.*, 2013), COLOX (gene expression of 29 genes) (Ciarloni *et al.*, 2016), and CANCERSEEK (16 genes + eight proteins) (Cohen *et al.*, 2018), recruited CRC cases in a complete or partial clinical setting and reported sensitivities of 89 %, 79.5%, and 64.9% at specificities of 90%, 90%, and 99.1%, respectively. However, the performance of these tests in screening settings remains unknown. When the performance of the blood‐based test COLOSENTRY (seven genes) was evaluated in screening setting, 61% sensitivity at 77% specificity for all stages was observed (Marshall *et al.*, 2010; Yip *et al.*, 2010). The FDA‐approved blood‐based test epi procolon 2.0 (Sept9 gene methylation) (Potter *et al.*, 2014) showed 59% sensitivity for early‐stage CRC at 79% specificity which is very close to the results observed in the validation sample of our study (61% sensitivity at 80% specificity). Nevertheless, the combination of the markers from tests such as COLODETECT, COLOX, and CANCERSEEK with the protein markers identified in the current study might potentially boost the diagnostic potential of blood‐based markers for early detection of CRC.

The 17 proteins that turned out in different algorithms as demonstrated here and in previous research are involved in different biological processes and mechanisms and interact directly in several pathways. Some of the identified proteins AREG, CEA, ITGAV, KRT19, MCP1, and TR were present in all three prediction algorithms. However, biomarkers such as MASP1, RARRES2, S100A4 and IL6, ITGA11, and TRAIL were specific for early and late stages, respectively. As presented in Table 4, the individual marker performances of these 17 markers for early and late stages in discovery and validation sets were heterogeneous. However, all the markers typically showed higher performances in discovery than validation sets. This once again highlights the differences in efficacy of markers in the clinically recruited and screening samples and in addition displays the need for external validation of signatures in screening colonoscopy cohorts. As described before and shown in Table 4, the markers that showed AUC ≥ 0.70 in both discovery and validation sets for early‐stage (AREG) and late‐stage CRC (CEA, KRT19, and TR) can be considered as strong potential protein markers for inclusion into multimarker signatures. Our current analysis and the previous studies (Chen *et al.*, 2017; Chen *et al.*, 2015) confirm that AREG has excellent diagnostic potential for the detection of CRC. Even though CEA has undisputed value for monitoring tumor recurrence and metastasis, its potential for early detection of CRC remains questionable (Lech *et al.*, 2013; Polat *et al.*, 2014). KRT19 and TR, which are both involved in biological processes of host–virus interaction, have seldom been found associated with early detection of CRC.

**Table 4:** Diagnostic performance of individual markers identified in signatures for detecting early‐ and late‐stage CRC in discovery and validation sets. Se, sensitivity; Sp, specificity.

Marker	Early stages (Stages I and II)												Late stages (Stages III and IV											
	Discovery set						Validation set						Discovery set						Validation set					
	*P*‐val	*P*‐val^adj^	AUC^* ^(95% CI)	AUC^BS ^(95% CI)	Se% at 80% Sp	Se% at 90% Sp	*P*‐val	*P*‐val^adj^	AUC^* ^(95% CI)	AUC^BS ^(95% CI)	Se% at 80% Sp	Se% at 90% Sp	*P*‐val	*P*‐val^adj^	AUC^*^ (95% CI)	AUC^BS^ (95% CI)	Se% at 80% Sp	Se% at 90% Sp	*P*‐val	*P*‐val^adj^	AUC^*^ (95% CI)	AUC^BS^ (95% CI)	Se% at 80% Sp	Se% at 90% Sp
AREG	<0.001	<0.001	0.7 (0.6–0.79)	0.68 (0.58–0.82)	45	35	<0.005	<0.01	0.73 (0.6–0.85)	0.7 (0.56–0.89)	55	36	<0.001	<0.001	0.87 (0.81–0.93)	0.86 (0.78–0.94)	78	65	<0.001	<0.001	0.71 (0.61–0.81)	0.69 (0.57–0.85)	49	33
CEA	<0.005	<0.005	0.67 (0.58–0.77)	0.64 (0.54–0.8)	42	27	<0.05	0.05	0.66 (0.51–0.81)	0.64 (0.46–0.85)	53	33	<0.001	<0.001	0.8 (0.71–0.88)	0.79 (0.67–0.91)	65	57	<0.001	<0.001	0.76 (0.65–0.87)	0.74 (0.6–0.91)	62	52
GZMB	<0.05	<0.05	0.63 (0.53–0.72)	0.59 (0.49–0.76)	28	12	0.06	0.11	0.63 (0.51–0.75)	0.55 (0.4–0.79)	25	12	<0.005	<0.005	0.66 (0.57–0.76)	0.64 (0.53–0.79)	33	12	0.65	0.65	0.53 (0.42–0.63)	0.45 (0.33–0.6)	14	7
IL6	<0.001	<0.005	0.68 (0.59–0.77)	0.65 (0.56–0.8)	39	17	<0.05	0.08	0.64 (0.52–0.76)	0.54 (0.32–0.79)	21	11	<0.001	<0.001	0.81 (0.74–0.89)	0.8 (0.71–0.91)	69	43	0.20	0.26	0.57 (0.47–0.68)	0.5 (0.32–0.69)	21	11
ITGA11	<0.001	<0.001	0.73 (0.65–0.82)	0.69 (0.58–0.84)	47	33	1.00	1.00	0.5 (0.38–0.63)	0.44 (0.29–0.6)	13	5	<0.001	<0.001	0.86 (0.79–0.92)	0.85 (0.75–0.93)	69	59	0.07	0.13	0.6 (0.5–0.7)	0.55 (0.44–0.72)	20	9
ITGAV	<0.001	<0.001	0.7 (0.61–0.79)	0.68 (0.56–0.81)	45	33	0.66	0.75	0.53 (0.4–0.66)	0.44 (0.28–0.64)	15	5	<0.001	<0.001	0.86 (0.79–0.93)	0.85 (0.76–0.94)	75	65	<0.005	<0.005	0.69 (0.59–0.78)	0.66 (0.56–0.81)	37	22
KRT19	<0.001	<0.005	0.68 (0.59–0.77)	0.66 (0.55–0.8)	42	25	<0.05	<0.05	0.68 (0.55–0.81)	0.64 (0.51–0.85)	44	30	<0.001	<0.001	0.84 (0.77–0.91)	0.83 (0.74–0.93)	67	60	<0.001	<0.001	0.74 (0.64–0.84)	0.72 (0.59–0.86)	52	39
MASP1	<0.005	<0.005	0.66 (0.57–0.75)	0.63 (0.53–0.77)	26	16	0.50	0.69	0.54 (0.41–0.68)	0.45 (0.26–0.65)	16	6	<0.01	<0.01	0.64 (0.54–0.74)	0.61 (0.51–0.78)	38	25	0.06	0.11	0.61 (0.5–0.72)	0.55 (0.39–0.75)	28	15
MCP1	<0.001	<0.005	0.69 (0.59–0.78)	0.66 (0.56–0.81)	42	24	0.66	0.75	0.53 (0.39–0.67)	0.44 (0.27–0.6)	14	8	<0.01	<0.01	0.64 (0.53–0.74)	0.61 (0.5–0.79)	40	28	0.18	0.26	0.58 (0.46–0.69)	0.5 (0.3–0.69)	22	12
NTproBNP	0.12	0.13	0.58 (0.47–0.68)	0.52 (0.34–0.69)	27	16	0.40	0.62	0.56 (0.43–0.68)	0.45 (0.28–0.64)	18	7	<0.001	<0.001	0.69 (0.6–0.79)	0.68 (0.58–0.82)	44	29	0.43	0.49	0.54 (0.42–0.67)	0.49 (0.31–0.68)	24	13
OPN	<0.005	<0.005	0.66 (0.56–0.75)	0.62 (0.53–0.78)	37	19	<0.005	<0.05	0.71 (0.6–0.82)	0.68 (0.56–0.86)	42	24	<0.001	<0.001	0.83 (0.76–0.9)	0.82 (0.73–0.92)	68	56	0.36	0.43	0.55 (0.44–0.67)	0.49 (0.32–0.67)	23	12
PON3	<0.001	<0.001	0.73 (0.64–0.81)	0.71 (0.6–0.84)	45	35	0.26	0.44	0.58 (0.45–0.7)	0.49 (0.3–0.71)	16	9	<0.001	<0.001	0.77 (0.69–0.86)	0.76 (0.66–0.89)	62	47	0.06	0.11	0.61 (0.5–0.71)	0.54 (0.34–0.73)	23	9
RARRES2	<0.001	<0.005	0.69 (0.6–0.78)	0.67 (0.56–0.81)	40	24	<0.05	<0.05	0.67 (0.57–0.78)	0.62 (0.53–0.81)	27	13	<0.001	<0.001	0.75 (0.66–0.84)	0.54 (0.18–0.8)	27	16	<0.05	0.08	0.62 (0.51–0.73)	0.54 (0.29–0.75)	28	11
S100A4	<0.005	<0.005	0.65 (0.56–0.74)	0.63 (0.51–0.78)	28	11	0.52	0.69	0.54 (0.42–0.66)	0.47 (0.32–0.67)	15	7	<0.005	<0.005	0.66 (0.56–0.75)	0.63 (0.53–0.78)	36	14	0.15	0.23	0.58 (0.47–0.69)	0.51 (0.29–0.69)	18	10
TR	<0.05	<0.05	0.62 (0.52–0.73)	0.6 (0.49–0.76)	38	29	<0.001	<0.01	0.74 (0.63–0.86)	0.72 (0.58–0.9)	48	35	<0.001	<0.001	0.76 (0.68–0.84)	0.75 (0.66–0.87)	53	43	<0.001	<0.001	0.74 (0.64–0.84)	0.71 (0.61–0.87)	50	28
TRAP	<0.05	<0.05	0.64 (0.53–0.74)	0.61 (0.49–0.77)	41	30	0.90	0.96	0.51 (0.39–0.63)	0.44 (0.31–0.58)	12	5	0.19	0.19	0.57 (0.46–0.67)	0.49 (0.31–0.67)	21	12	0.65	0.65	0.53 (0.42–0.64)	0.44 (0.31–0.6)	17	7
TRAIL	0.26	0.26	0.56 (0.45–0.66)	0.51 (0.35–0.68)	24	16	<0.05	0.05	0.65 (0.54–0.77)	0.59 (0.46–0.8)	26	12	<0.001	<0.001	0.75 (0.67–0.83)	0.74 (0.64–0.86)	48	30	<0.005	<0.05	0.67 (0.56–0.77)	0.63 (0.51–0.8)	34	18

Precursors of CRC, that is, AA, are at a risk of developing into invasive CRC over time. The detection of these precursors and their timely removal could contribute substantially to reduction in CRC risk. Detecting these noninvasive precursors with blood‐based tests is likely to be difficult. In the current study, the failure of early‐stage algorithm (that was evaluated for AA participants) is in line with results of the few studies validating diagnostic performance of blood‐based tests in true screening setting, such as the PRESEPT clinical trial on Sept9 gene methylation (Church *et al.*, 2014; Potter *et al.*, 2014). Possible combinations of protein biomarkers obtained with rigorous validation (as in the current study) with other types of genomic, epigenetic, or metabolomic biomarkers may bolster the diagnostic potential of blood‐based tests for early detection of CRC and its precursors.

A major strength of the current study is that we used a two‐stage design, with validation performed exclusively in a true screening setting. However, despite the overall very large size of the BLITZ study (*N* = 9245) the number of participants with CRC was still rather limited, a feature that is common for screening settings. Using cutting‐edge statistical machine learning algorithms, 275 markers were simultaneously evaluated for possible combinations and comparisons of the tested markers. In addition to an algorithm for overall prediction of CRC presence, signatures for stage‐specific detection were derived and internal as well as external validation was performed. For early stage in external independent validation, sensitivity of 61% was observed at 80% specificity, which is comparable to DNA‐Epi proColon 2.0, the only FDA‐approved blood‐based test for CRC detection. Major limitations include the limited sample size of CRC patients, especially for stage‐specific analyses, leading to rather wide CIs of the derived indicators of diagnostic performance. Given that no other cancers were included in our study, we could not evaluate whether and to what extent the identified markers are CRC‐specific. Therefore, further investigation with patients suffering from other cancers or systemic diseases would be essential. Further research including larger numbers of CRC cases should also address potential differences in detection of colon and rectum cancer. Furthermore, even though the reproducibility of PEAs is better compared with mass spectrometry‐based methods (Smith and Gerszten, 2017), replication of our findings using other quantification techniques should be aimed for. Additionally, further investigation should address to what extent the 17 identified proteins are secreted *in vitro* by tumor cells or immune cells.

## 5 Conclusion

We have identified several proteins that individually and in combination carry diagnostic potential for the detection of CRC. With 61% sensitivity at 80% specificity in a true screening setting, diagnostic performance of a 12‐marker algorithm was comparable to diagnostic performance of DNA‐Epi proColon 2.0, the only FDA‐approved blood‐based CRC screening test. Although not competitive with the best available stool‐based tests, the combination of identified protein markers with other informative blood‐based markers could contribute to the development of a promising blood‐based test for CRC screening.

## Conflict of interest

The German Cancer Research Center has received industrial grants related to blood markers for early detection of CRC from Epigenomics, Applied Proteomics, and Roche Diagnostics.

## Author contributions

HB conceived and supervised the studies. KT, KW, and HB coordinated the studies. MB planned and coordinated this project, selected and shipped the samples, conducted the statistical analyses, interpreted the results, and drafted the manuscript. MB, KW, KT, AB, PS‐K, and HB critically reviewed the manuscript, contributed to its revision, and approved the final version.

## Supporting information


**Fig. S1.** STARD showing selection of study participants enrolled in the iDa Study.
**Fig. S2.** STARD showing selection of study participants enrolled in the ASTER Study during 2013–2016.
**Fig. S3.** Experimental workflow of the study.
**Fig. S4.** Interaction of identified markers from all predictor models in canonical pathways at subcellular level.
**Fig. S5.** Involvement of identified markers from all predictor models in different organ toxicities.

**Table S1.** List of 276 proteins measured in the three Olink Multiplex Panels.

**Table S2.** Diagnostic performance of all 275 proteins markers in discovery and validation set for all‐stage CRC detection.

**Table S3.** The algorithms identified from the discovery set for (all/early/late) stage CRC detection.

**Table S4.** Diagnostic performance of the individual markers for detection of CRC in discovery and validation sets stratified by cancer location.

**Table S5.** Diagnostic performance of the 9‐marker signature for detection of CRC in discovery and validation sets stratified by cancer location.
