# Supplementary material for: Multiplex screening of 275 plasma protein biomarkers to identify a signature for early detection of colorectal cancer
Source: Mol Oncol. 2019 Nov 13;14(1):8–21. doi: 10.1002/1878-0261.12591 (PMC6944100; doi:10.1002/1878-0261.12591)
Supplement: Supplementary file 1 — Fig. S1. STARD showing selection of study participants enrolled in the iDa Study. Fig. S2. STARD showing selection of study participants enrolled in the ASTER Study during 2013–2016. Fig. S3. Experimental workflow of the study. Fig. S4. Interaction of identified markers from all predictor models in canonical pathways at subcellular level. Fig. S5. Involvement of identified markers from all predictor models in different organ toxicities. [file MOL2-14-8-s001.docx]

**
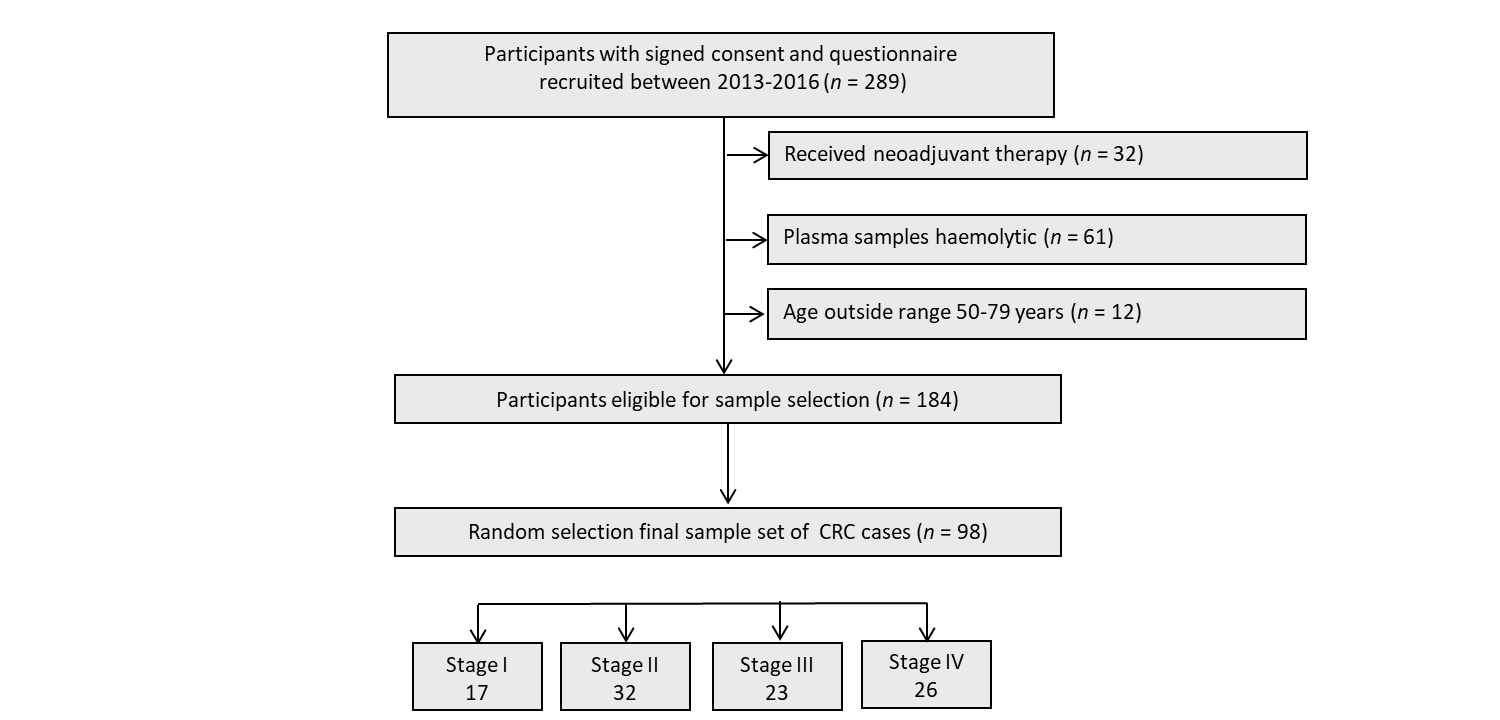
**

**Supplementary Figure 1:** STARD (Standards for Reporting of Diagnostic Accuracy) showing selection of study participants enrolled in the iDa Study.

**Abbreviation: CRC**- colorectal cancer.


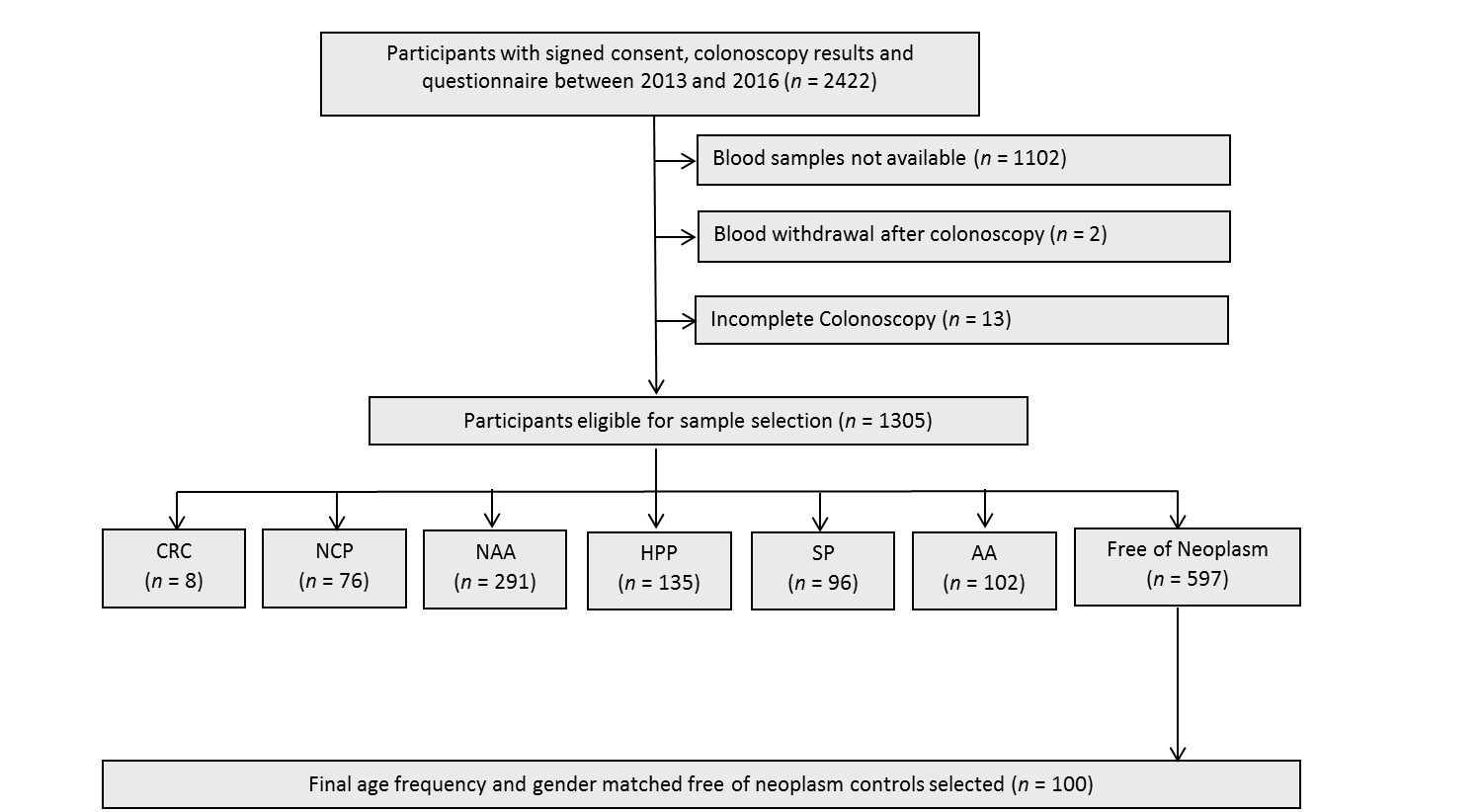


**Supplementary Figure 2:** STARD (Standards for Reporting of Diagnostic Accuracy) showing selection of study participants enrolled in the ASTER Study during 2013-2016.

**Abbreviations: AA-** advanced adenoma; **CRC**- colorectal cancer; **NAA**- non-advanced adenoma; **NCP**- non-classified polyp.


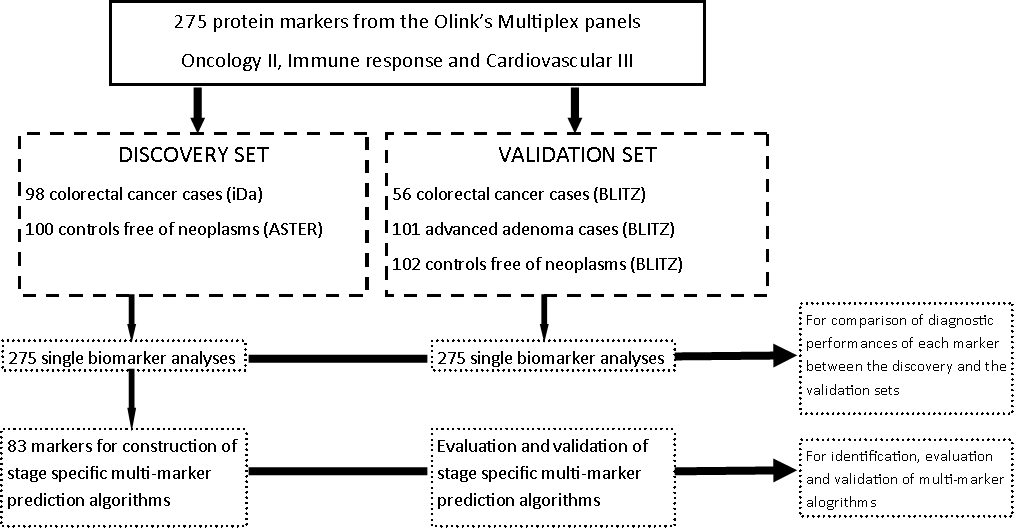


**Supplementary Figure 3:** Experimental workflow of the study.

**
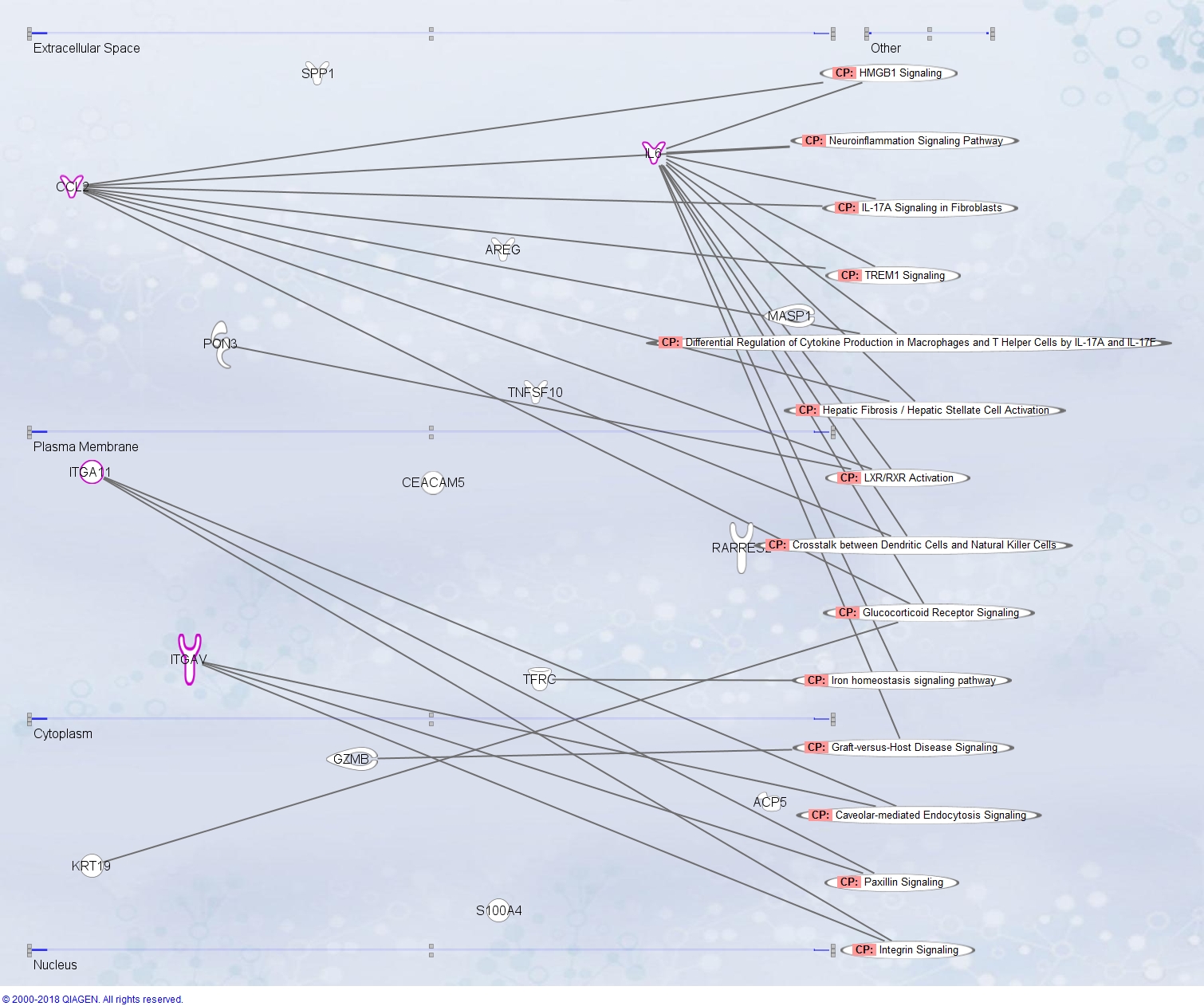
**

**Supplementary Figure 4:** Interaction of identified markers from all predictor models in canonical pathways at sub-cellular level.

**All proteins abbreviations:** **AREG**- amphiregulin; **CEA**- carcinoembryonic antigen; **GZMB**- granzyme B; **IL6**- interleukin-6; **ITGA11**- integrin alpha 11; **ITGAV**- integrin alpha V; **KRT19**- keratin, type I cytoskeletal 19; **MASP1**- mannan-binding lectin serine protease 1 ; **MCP1**- monocyte chemotactic protein 1; **NTproBNP**- n terminal prohormone brain natriuretic peptide; **OPN**- osteopontin; **PON3**- paraoxonase 3; **RARRES2**- retinoic acid receptor responder protein 2; **S100A4**- protein S100-A4; **TR**- transferrin receptor protein 1; **TRAP**- tartrate-resistant acid phosphatase type 5; **TRAIL-** tnf related apoptosis inducing ligand.

**
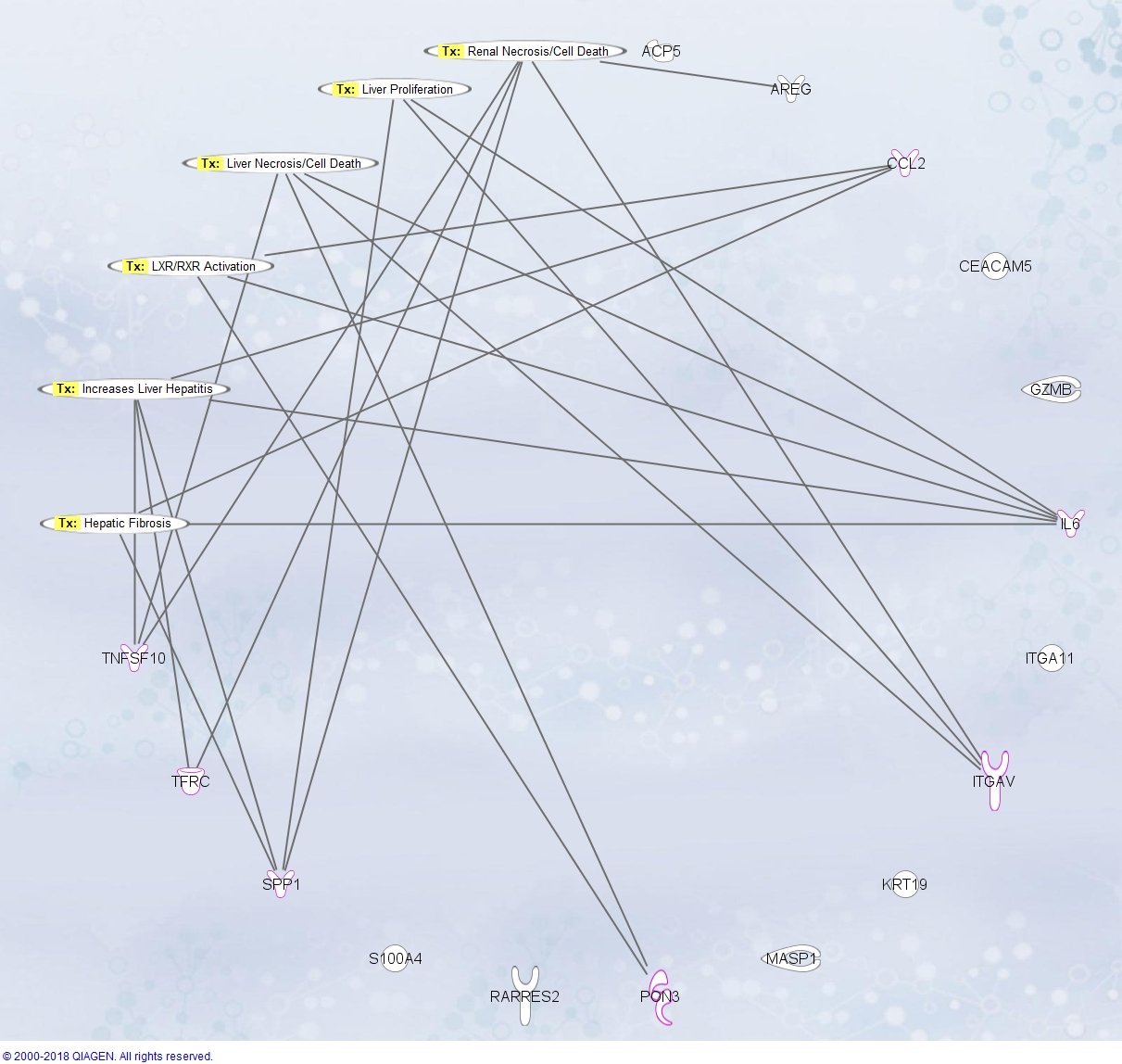
**

**Supplementary Figure 5:** Involvement of identified markers from all predictor models in different organ toxicities.

**All proteins abbreviations:** **AREG**- amphiregulin; **CEA**- carcinoembryonic antigen; **GZMB**- granzyme B; **IL6**- interleukin-6; **ITGA11**- integrin alpha 11; **ITGAV**- integrin alpha V; **KRT19**- keratin, type I cytoskeletal 19; **MASP1**- mannan-binding lectin serine protease 1 ; **MCP1**- monocyte chemotactic protein 1; **NTproBNP**- n terminal prohormone brain natriuretic peptide; **OPN**- osteopontin; **PON3**- paraoxonase 3; **RARRES2**- retinoic acid receptor responder protein 2; **S100A4**- protein S100-A4; **TR**- transferrin receptor protein 1; **TRAP**- tartrate-resistant acid phosphatase type 5; **TRAIL-** tnf related apoptosis inducing ligand.
