# Supplementary material for: Multiplex screening of 275 plasma protein biomarkers to identify a signature for early detection of colorectal cancer
Source: Mol Oncol. 2019 Nov 13;14(1):8–21. doi: 10.1002/1878-0261.12591 (PMC6944100; doi:10.1002/1878-0261.12591)
Supplement: Supplementary file 2 — Table S1. List of 276 proteins measured in the three Olink Multiplex Panels. [file MOL2-14-8-s002.docx]

**Supplementary Table 1:** List of 276 proteins measured in the three Olink Multiplex Panels

| Protein | Uniprot ID | Panel |
| --- | --- | --- |
| Aminopeptidase N (AP-N) | P15144 | Cardiovascular III |
| Azurocidin (AZU1) | P20160 | Cardiovascular III |
| Bleomycin hydrolase (BLM hydrolase) | Q13867 | Cardiovascular III |
| Cadherin-5 (CDH5) | P33151 | Cardiovascular III |
| Carboxypeptidase A1 (CPA1) | P15085 | Cardiovascular III |
| Carboxypeptidase B (CPB1) | P15086 | Cardiovascular III |
| Caspase-3 (CASP-3) | P42574 | Cardiovascular III |
| Cathepsin D (CTSD) | P07339 | Cardiovascular III |
| Cathepsin Z (CTSZ) | Q9UBR2 | Cardiovascular III |
| C-C motif chemokine 15 (CCL15) | Q16663 | Cardiovascular III |
| C-C motif chemokine 16 (CCL16) | O15467 | Cardiovascular III |
| C-C motif chemokine 24 (CCL24) | O00175 | Cardiovascular III |
| CD166 antigen (ALCAM) | Q13740 | Cardiovascular III |
| Chitinase-3-like protein 1 (CHI3L1) | P36222 | Cardiovascular III |
| Chitotriosidase-1 (CHIT1) | Q13231 | Cardiovascular III |
| Collagen alpha-1(I) chain (COL1A1) | P02452 | Cardiovascular III |
| Complement component C1q receptor (CD93) | Q9NPY3 | Cardiovascular III |
| Contactin-1 (CNTN1) | Q12860 | Cardiovascular III |
| C-X-C motif chemokine 16 (CXCL16) | Q9H2A7 | Cardiovascular III |
| Cystatin-B (CSTB) | P04080 | Cardiovascular III |
| Elafin (PI3) | P19957 | Cardiovascular III |
| Ephrin type-B receptor 4 (EPHB4) | P54760 | Cardiovascular III |
| Epidermal growth factor receptor (EGFR ) | P00533 | Cardiovascular III |
| Epithelial cell adhesion molecule (Ep-CAM) | P16422 | Cardiovascular III |
| E-selectin (SELE) | P16581 | Cardiovascular III |
| Fatty acid-binding protein, adipocyte (FABP4) | P15090 | Cardiovascular III |
| Galectin-3 (Gal-3) | P17931 | Cardiovascular III |
| Galectin-4 (Gal-4) | P56470 | Cardiovascular III |
| Granulins (GRN) | P28799 | Cardiovascular III |
| Growth/differentiation factor 15 (GDF-15) | Q99988 | Cardiovascular III |
| Human GPVI Antibody (GPVI) | Q9HCN6 | Cardiovascular III |
| Insulin-like growth factor-binding protein 1 (IGFBP-1) | P08833 | Cardiovascular III |
| Insulin-like growth factor-binding protein 2 (IGFBP-2) | P18065 | Cardiovascular III |
| Insulin-like growth factor-binding protein 7 (IGFBP-7) | Q16270 | Cardiovascular III |
| Integrin beta-2 (ITGB2) | P05107 | Cardiovascular III |
| Intercellular adhesion molecule 2 (ICAM-2) | P13598 | Cardiovascular III |
| Interleukin-1 receptor type 1 (IL-1RT1) | P14778 | Cardiovascular III |
| Interleukin-1 receptor type 2 (IL-1RT2) | P27930 | Cardiovascular III |
| Interleukin-17 receptor A (IL-17RA) | Q96F46 | Cardiovascular III |
| Interleukin-18-binding protein (IL-18BP) | O95998 | Cardiovascular III |
| Interleukin-2 receptor subunit alpha (IL2-RA) | P01589 | Cardiovascular III |
| Interleukin-6 receptor subunit alpha (IL-6RA) | P08887 | Cardiovascular III |
| Junctional adhesion molecule A (JAM-A) | Q9Y624 | Cardiovascular III |
| Protein | **Uniprot ID** | **Panel** |
| Kallikrein-6 (KLK6 ) | Q92876 | Cardiovascular III |
| Low-density lipoprotein receptor (LDL receptor) | P01130 | Cardiovascular III |
| Lymphotoxin-beta receptor (LTBR) | P36941 | Cardiovascular III |
| Matrix extracellular phosphoglycoprotein (MEPE) | Q9NQ76 | Cardiovascular III |
| Matrix metalloproteinase-2 (MMP-2) | P08253 | Cardiovascular III |
| Matrix metalloproteinase-3 (MMP-3) | P08254 | Cardiovascular III |
| Matrix metalloproteinase-9 (MMP-9) | P14780 | Cardiovascular III |
| Metalloproteinase inhibitor 4 (TIMP4) | Q99727 | Cardiovascular III |
| Monocyte chemotactic protein 1 (MCP-1) | P13500 | Cardiovascular III |
| Myeloblastin (PRTN3) | P24158 | Cardiovascular III |
| Myeloperoxidase (MPO) | P05164 | Cardiovascular III |
| Myoglobin (MB) | P02144 | Cardiovascular III |
| Neurogenic locus notch homolog protein 3 (Notch 3) | Q9UM47 | Cardiovascular III |
| N-terminal prohormone brain natriuretic peptide (NT-proBNP) | NA | Cardiovascular III |
| Osteopontin (OPN) | P10451 | Cardiovascular III |
| Osteoprotegerin (OPG) | O00300 | Cardiovascular III |
| Paraoxonase (PON3) | Q15166 | Cardiovascular III |
| Peptidoglycan recognition protein 1 (PGLYRP1) | O75594 | Cardiovascular III |
| Perlecan (PLC) | P98160 | Cardiovascular III |
| Plasminogen activator inhibitor 1 (PAI) | P05121 | Cardiovascular III |
| Platelet endothelial cell adhesion molecule (PECAM-1) | P16284 | Cardiovascular III |
| Platelet-derived growth factor subunit A (PDGF subunit A) | P04085 | Cardiovascular III |
| Proprotein convertase subtilisin/kexin type 9 (PCSK9) | Q8NBP7 | Cardiovascular III |
| Protein delta homolog 1 (DLK-1) | P80370 | Cardiovascular III |
| P-selectin (SELP) | P16109 | Cardiovascular III |
| Pulmonary surfactant-associated protein D (PSP-D) | P35247 | Cardiovascular III |
| Resistin (RETN) | Q9HD89 | Cardiovascular III |
| Retinoic acid receptor responder protein 2 (RARRES2) | Q99969 | Cardiovascular III |
| Scavenger receptor cysteine-rich type 1 protein M130 (CD163) | Q86VB7 | Cardiovascular III |
| Secretoglobin family 3A member 2 (SCGB3A2) | Q96PL1 | Cardiovascular III |
| Spondin-1 (SPON1) | Q9HCB6 | Cardiovascular III |
| ST2 protein (ST2) | Q01638 | Cardiovascular III |
| Tartrate-resistant acid phosphatase type 5 (TR-AP) | P13686 | Cardiovascular III |
| Tissue factor pathway inhibitor (TFPI) | P10646 | Cardiovascular III |
| Tissue-type plasminogen activator (t-PA) | P00750 | Cardiovascular III |
| Transferrin receptor protein 1 (TR) | P02786 | Cardiovascular III |
| Trefoil factor 3 (TFF3) | Q07654 | Cardiovascular III |
| Trem-like transcript 2 protein (TLT-2) | Q5T2D2 | Cardiovascular III |
| Tumor necrosis factor ligand superfamily member 13B (TNFSF13B) | Q9Y275 | Cardiovascular III |
| Tumor necrosis factor receptor 1 (TNF-R1) | P19438 | Cardiovascular III |
| Tumor necrosis factor receptor 2 (TNF-R2) | P20333 | Cardiovascular III |
| Tumor necrosis factor receptor superfamily member 10C (TNFRSF10C) | O14798 | Cardiovascular III |
| Tumor necrosis factor receptor superfamily member 14 (TNFRSF14) | Q92956 | Cardiovascular III |
| Tumor necrosis factor receptor superfamily member 6 (FAS ) | P25445 | Cardiovascular III |
| Tyrosine-protein kinase receptor UFO (AXL) | P30530 | Cardiovascular III |
| Protein | **Uniprot ID** | **Panel** |
| Tyrosine-protein phosphatase non-receptor type substrate 1 (SHPS-1) | P78324 | Cardiovascular III |
| Urokinase plasminogen activator surface receptor (U-PAR) | Q03405 | Cardiovascular III |
| Urokinase-type plasminogen activator (uPA) | P00749 | Cardiovascular III |
| von Willebrand factor (vWF) | P04275 | Cardiovascular III |
| Allergin-1 (MILR1) | Q7Z6M3 | Immune response |
| Amphiregulin (AREG) | P15514 | Immune response |
| Aryl hydrocarbon receptor nuclear translocator (ARNT) | P27540 | Immune response |
| Baculoviral IAP repeat-containing protein 2 (BIRC2) | Q13490 | Immune response |
| Beta-galactosidase (GLB1) | P16278 | Immune response |
| Butyrophilin subfamily 3 member A2 (BTN3A2) | P78410 | Immune response |
| CD83 antigen (CD83) | Q01151 | Immune response |
| Contactin-associated protein-like 2 (CNTNAP2) | Q9UHC6 | Immune response |
| Corneodesmosin (CDSN) | Q15517 | Immune response |
| Corticosteroid 11-beta-dehydrogenase isozyme 1 (HSD11B1) | P28845 | Immune response |
| Coxsackievirus and adenovirus receptor (CXADR) | P78310 | Immune response |
| C-type lectin domain family 4 member A (CLEC4A) | Q9UMR7 | Immune response |
| C-type lectin domain family 4 member C (CLEC4C) | Q8WTT0 | Immune response |
| C-type lectin domain family 4 member D (CLEC4D) | Q8WXI8 | Immune response |
| C-type lectin domain family 4 member G (CLEC4G) | Q6UXB4 | Immune response |
| C-type lectin domain family 6 member A (CLEC6A) | Q6EIG7 | Immune response |
| C-type lectin domain family 7 member A (CLEC7A) | Q9BXN2 | Immune response |
| Cytoskeleton-associated protein 4 (CKAP4) | Q07065 | Immune response |
| Diacylglycerol kinase zeta (DGKZ) | Q13574 | Immune response |
| Discoidin, CUB and LCCL domain-containing protein 2 (DCBLD2) | Q96PD2 | Immune response |
| DNA fragmentation factor subunit alpha (DFFA) | O00273 | Immune response |
| Dual adapter for phosphotyrosine and 3-phosphotyrosine and 3-phosphoinositide (DAPP1) | Q9UN19 | Immune response |
| Dynactin subunit 1 (DCTN1) | Q14203 | Immune response |
| E3 ubiquitin-protein ligase TRIM21 (TRIM21) | P19474 | Immune response |
| Egl nine homolog 1 (EGLN1) | Q9GZT9 | Immune response |
| Eotaxin (CCL11) | P51671 | Immune response |
| Eukaryotic translation initiation factor 4 gamma 1 (EIF4G1) | Q04637 | Immune response |
| Eukaryotic translation initiation factor 5A-1 (EIF5A) | P63241 | Immune response |
| Fc receptor-like protein 3 (FCRL3) | Q96P31 | Immune response |
| Fc receptor-like protein 6 (FCRL6) | Q6DN72 | Immune response |
| Fibroblast growth factor 2 (FGF2) | P09038 | Immune response |
| FXYD domain-containing ion transport regulator 5 (FXYD5) | Q96DB9 | Immune response |
| Hematopoietic lineage cell-specific protein (HCLS1) | P14317 | Immune response |
| Histamine N-methyltransferase (HNMT) | P50135 | Immune response |
| Importin subunit alpha-5 (KPNA1) | P52294 | Immune response |
| Inactive dipeptidyl peptidase 10 (DPP10) | Q8N608 | Immune response |
| Integral membrane protein 2A (ITM2A) | O43736 | Immune response |
| Integrin alpha-11 (ITGA11) | Q9UKX5 | Immune response |
| Integrin alpha-6 (ITGA6) | P23229 | Immune response |
| Integrin beta-6 (ITGB6) | P18564 | Immune response |
| Protein | **Uniprot ID** | **Panel** |
| Interferon lambda receptor 1 (IFNLR1) | Q8IU57 | Immune response |
| Interferon regulatory factor 9 (IRF9) | Q00978 | Immune response |
| Interleukin-1 receptor-associated kinase 1 (IRAK1) | P51617 | Immune response |
| Interleukin-1 receptor-associated kinase 4 (IRAK4) | Q9NWZ3 | Immune response |
| Interleukin-10 (IL10) | P22301 | Immune response |
| Interleukin-12 receptor subunit beta-1 (IL12RB1) | P42701 | Immune response |
| Interleukin-5 (IL5) | P05113 | Immune response |
| Interleukin-6 (IL6) | P05231 | Immune response |
| Islet cell autoantigen 1 (ICA1) | Q05084 | Immune response |
| Keratin, type I cytoskeletal 19 (KRT19) | P08727 | Immune response |
| Leukocyte immunoglobulin-like receptor subfamily B member 4 (LILRB4) | Q8NHJ6 | Immune response |
| Lymphocyte activation gene 3 protein (LAG3) | P18627 | Immune response |
| Lymphocyte antigen 75 (LY75) | O60449 | Immune response |
| Lysosome-associated membrane glycoprotein 3 (LAMP3) | Q9UQV4 | Immune response |
| Mannan-binding lectin serine protease 1 (MASP1) | P48740 | Immune response |
| Merlin (NF2) | P35240 | Immune response |
| Methylated-DNA--protein-cysteine methyltransferase (MGMT) | P16455 | Immune response |
| Natural cytotoxicity triggering receptor 1 (NCR1) | O76036 | Immune response |
| Natural killer cells antigen CD94 (KLRD1) | Q13241 | Immune response |
| Neurabin-2 (PPP1R9B) | Q96SB3 | Immune response |
| Neurotrophin-4 (NTF4) | P34130 | Immune response |
| Nuclear factor of activated T-cells, cytoplasmic 3 (NFATC3) | Q12968 | Immune response |
| Parathyroid hormone/parathyroid hormone-related peptide receptor (PTH1R) | Q03431 | Immune response |
| PC4 and SFRS1-interacting protein (PSIP1) | O75475 | Immune response |
| Peroxiredoxin-1 (PRDX1) | Q06830 | Immune response |
| Peroxiredoxin-5, mitochondrial (PRDX5) | P30044 | Immune response |
| Phosphoinositide 3-kinase adapter protein 1 (PIK3AP1) | Q6ZUJ8 | Immune response |
| Plexin-A4 (PLXNA4) | Q9HCM2 | Immune response |
| Polypeptide N-acetylgalactosaminyltransferase 3 (GALNT3) | Q14435 | Immune response |
| Probable ATP-dependent RNA helicase DDX58 (DDX58) | O95786 | Immune response |
| Protein FAM3B (FAM3B) | P58499 | Immune response |
| Protein HEXIM1 (HEXIM1) | O94992 | Immune response |
| Protein kinase C theta type (PRKCQ) | Q04759 | Immune response |
| Protein sprouty homolog 2 (SPRY2) | O43597 | Immune response |
| Protein-arginine deiminase type-2 (PADI2) | Q9Y2J8 | Immune response |
| SH2 domain-containing protein 1A (SH2D1A) | O60880 | Immune response |
| SH2B adapter protein 3 (SH2B3) | Q9UQQ2 | Immune response |
| Signaling threshold-regulating transmembrane adapter 1 (SIT1) | Q9Y3P8 | Immune response |
| SRSF protein kinase 2 (SRPK2) | P78362 | Immune response |
| Stanniocalcin-1 (STC1) | P52823 | Immune response |
| Stromal cell-derived factor 1 (CXCL12) | P48061 | Immune response |
| T-cell-specific surface glycoprotein CD28 (CD28) | P10747 | Immune response |
| Thioredoxin-dependent peroxide reductase, mitochondrial (PRDX3) | P30048 | Immune response |
| Protein | **Uniprot ID** | **Panel** |
| TNF receptor-associated factor 2 (TRAF2) | Q12933 | Immune response |
| TRAF family member-associated NF-kappa-B activator (TANK) | Q92844 | Immune response |
| Transcription factor AP-1 (JUN) | P05412 | Immune response |
| Transcription regulator protein BACH1 (BACH1) | O14867 | Immune response |
| Triggering receptor expressed on myeloid cells 1 (TREM1) | Q9NP99 | Immune response |
| Tripartite motif-containing protein 5 (TRIM5) | Q9C035 | Immune response |
| Tryptase alpha/beta-1 (TPSAB1) | Q15661 | Immune response |
| Tumor necrosis factor receptor superfamily member EDAR (EDAR) | Q9UNE0 | Immune response |
| Zinc finger and BTB domain-containing protein 16 (ZBTB16) | Q05516 | Immune response |
| 5'-nucleotidase (5'-NT) | P21589 | Oncology II |
| A disintegrin and metalloproteinase with thrombospondin motifs 15 (ADAM-TS 15) | Q8TE58 | Oncology II |
| Alpha-taxilin (TXLNA) | P40222 | Oncology II |
| Amphiregulin (AREG) | P15514 | Oncology II |
| Annexin A1 (ANXA1) | P04083 | Oncology II |
| Carbonic anhydrase IX (CAIX) | Q16790 | Oncology II |
| Carboxypeptidase E (CPE) | P16870 | Oncology II |
| Carcinoembryonic antigen (CEA) | P06731 | Oncology II |
| Carcinoembryonic antigen-related cell adhesion molecule 1 (CEACAM1) | P13688 | Oncology II |
| Cathepsin L2 (CTSV) | O60911 | Oncology II |
| CD160 antigen (CD160) | O95971 | Oncology II |
| CD27 antigen (CD27) | P26842 | Oncology II |
| CD48 antigen (CD48) | P09326 | Oncology II |
| CD70 antigen (CD70) | P32970 | Oncology II |
| Cornulin (CRNN) | Q9UBG3 | Oncology II |
| C-type lectin domain family 4 member K (CD207) | Q9UJ71 | Oncology II |
| C-X-C motif chemokine 13 (CXCL13 ) | O43927 | Oncology II |
| Cyclin-dependent kinase inhibitor 1 (CDKN1A) | P38936 | Oncology II |
| Delta-like protein 1 (DLL1) | O00548 | Oncology II |
| Disintegrin and metalloproteinase domain-containing protein 8 (ADAM8) | P78325 | Oncology II |
| Endothelial cell-specific molecule 1 (ESM-1) | Q9NQ30 | Oncology II |
| Ephrin type-A receptor 2 (EPHA2) | P29317 | Oncology II |
| Fas antigen ligand (FasL) | P48023 | Oncology II |
| FAS-associated death domain protein (FADD) | Q13158 | Oncology II |
| Fc receptor-like B (FCRLB) | Q6BAA4 | Oncology II |
| Fibroblast growth factor-binding protein 1 (FGF-BP1) | Q14512 | Oncology II |
| Folate receptor alpha (FR-alpha) | P15328 | Oncology II |
| Folate receptor gamma (FR-gamma) | P41439 | Oncology II |
| Furin (FUR) | P09958 | Oncology II |
| Galectin-1 (Gal-1) | P09382 | Oncology II |
| Glypican-1 (GPC1) | P35052 | Oncology II |
| Granzyme B (GZMB) | P10144 | Oncology II |
| Granzyme H (GZMH) | P20718 | Oncology II |
| Hepatocyte growth factor (HGF) | P14210 | Oncology II |
| Protein | **Uniprot ID** | **Panel** |
| ICOS ligand (ICOSLG) | O75144 | Oncology II |
| Insulin-like growth factor 1 receptor (IGF1R) | P08069 | Oncology II |
| Integrin alpha-V (ITGAV) | P06756 | Oncology II |
| Integrin beta-5 (ITGB5) | P18084 | Oncology II |
| Interferon gamma receptor 1 (IFN-gamma-R1) | P15260 | Oncology II |
| Interleukin-6 (IL6) | P05231 | Oncology II |
| Kallikrein-11 (hK11) | Q9UBX7 | Oncology II |
| Kallikrein-13 (KLK13) | Q9UKR3 | Oncology II |
| Kallikrein-14 (hK14) | Q9P0G3 | Oncology II |
| Kallikrein-8 (hK8) | O60259 | Oncology II |
| Ly6/PLAUR domain-containing protein 3 (LYPD3) | O95274 | Oncology II |
| Melanoma-derived growth regulatory protein (MIA) | Q16674 | Oncology II |
| Mesothelin (MSLN) | Q13421 | Oncology II |
| Methionine aminopeptidase 2 (MetAP 2) | P50579 | Oncology II |
| MHC class I polypeptide-related sequence A/B (MIC-A/B) | Q29983,Q29980 | Oncology II |
| Midkine (MK) | P21741 | Oncology II |
| Mothers against decapentaplegic homolog 5 (MAD homolog 5) | Q99717 | Oncology II |
| Mucin-16 (MUC-16) | Q8WXI7 | Oncology II |
| Nectin-4 (PVRL4) | Q96NY8 | Oncology II |
| Pancreatic prohormone (PPY) | P01298 | Oncology II |
| Podocalyxin (PODXL) | O00592 | Oncology II |
| Pro-epidermal growth factor (EGF) | P01133 | Oncology II |
| Protein CYR61 (CYR61) | O00622 | Oncology II |
| Protein S100-A11 (S100A11) | P31949 | Oncology II |
| Protein S100-A4 (S100A4) | P26447 | Oncology II |
| Proto-oncogene tyrosine-protein kinase receptor Ret (RET) | P07949 | Oncology II |
| Receptor tyrosine-protein kinase erbB-2 (ErbB2/HER2) | P04626 | Oncology II |
| Receptor tyrosine-protein kinase erbB-3 (ErbB3/HER3) | P21860 | Oncology II |
| Receptor tyrosine-protein kinase erbB-4 (ErbB4/HER4) | Q15303 | Oncology II |
| R-spondin-3 (RSPO3) | Q9BXY4 | Oncology II |
| Secretory carrier-associated membrane protein 3 (SCAMP3) | O14828 | Oncology II |
| Seizure 6-like protein (SEZ6L) | Q9BYH1 | Oncology II |
| SPARC (SPARC) | P09486 | Oncology II |
| Stem cell factor (SCF) | P21583 | Oncology II |
| Syndecan-1 (SYND1) | P18827 | Oncology II |
| T-cell leukemia / lymphoma protein 1A (TCL1A) | P56279 | Oncology II |
| TGF-beta receptor type-2 (TGFR-2) | P37173 | Oncology II |
| Tissue factor pathway inhibitor 2 (TFPI-2) | P48307 | Oncology II |
| T-lymphocyte surface antigen Ly-9 (LY9) | Q9HBG7 | Oncology II |
| TNF-related apoptosis-inducing ligand (TRAIL) | P50591 | Oncology II |
| Toll-like receptor 3 (TLR3) | O15455 | Oncology II |
| Transforming growth factor alpha (TGF-alpha) | P01135 | Oncology II |
| Transmembrane glycoprotein NMB (GPNMB) | Q14956 | Oncology II |
| Tumor necrosis factor ligand superfamily member 13 (TNFSF13) | O75888 | Oncology II |
| Tumor necrosis factor receptor superfamily member 19 (TNFRSF19) | Q9NS68 | Oncology II |
| Protein | **Uniprot ID** | **Panel** |
| Tumor necrosis factor receptor superfamily member 4 (TNFRSF4 ) | P43489 | Oncology II |
| Tumor necrosis factor receptor superfamily member 6B (TNFRSF6B) | O95407 | Oncology II |
| Tyrosine-protein kinase ABL1 (ABL1) | P00519 | Oncology II |
| Tyrosine-protein kinase Lyn (LYN) | P07948 | Oncology II |
| Vascular endothelial growth factor A (VEGF-A) | P15692 | Oncology II |
| Vascular endothelial growth factor receptor 2 (VEGFR-2) | P35968 | Oncology II |
| Vascular endothelial growth factor receptor 3 (VEGFR-3) | P35916 | Oncology II |
| VEGF-co regulated chemokine 1 (CXL17) | Q6UXB2 | Oncology II |
| Vimentin (VIM) | P08670 | Oncology II |
| WAP four-disulfide core domain protein 2 (WFDC2) | Q14508 | Oncology II |
| Wnt inhibitory factor 1 (WIF-1) | Q9Y5W5 | Oncology II |
| WNT1-inducible-signaling pathway protein 1 (WISP-1) | O95388 | Oncology II |
| Xaa-Pro aminopeptidase 2 (XPNPEP2) | O43895 | Oncology II |
