# Supplementary material for: Multiplex screening of 275 plasma protein biomarkers to identify a signature for early detection of colorectal cancer
Source: Mol Oncol. 2019 Nov 13;14(1):8–21. doi: 10.1002/1878-0261.12591 (PMC6944100; doi:10.1002/1878-0261.12591)
Supplement: Supplementary file 3 — Table S2. Diagnostic performance of all 275 proteins markers in discovery and validation set for all‐stage CRC detection. [file MOL2-14-8-s003.docx]

**Supplementary Table 2:** Diagnostic performance of all 275 proteins markers in discovery and validation set for all stage CRC detection

|  | DISCOVERY SET | | | | | | | | | VALIDATION SET | | | | | | | | |
| --- | --- | --- | --- | --- | --- | --- | --- | --- | --- | --- | --- | --- | --- | --- | --- | --- | --- | --- |
| Marker | **CRC_**  **median** | **Control_**  **median** | **Fold Change** | **p_value** | **p_value^adj^** | **AUC^*^**  **(95% CI)** | **AUC^BS^**  **(95% CI)** | **Se^BS^ % at**  **80% Sp** | **Se^BS^ % at**  **90% Sp** | **CRC_**  **median** | **Control_**  **median** | **Fold Change** | **p_value** | **p_value^adj^** | **AUC^*^**  **(95% CI)** | **AUC^BS^**  **(95% CI)** | **Se^BS^ % at**  **80% Sp** | **Se^BS^ % at**  **90% Sp** |
| ABL1 | 4.06 | 4.07 | -0.02 | 0.93 | 0.95 | 0.5[0.41-0.58] | 0.46[0.37-0.57] | 16% | 8% | 4.33 | 3.80 | 1.07 | 0.08 | 0.23 | 0.58[0.49-0.67] | 0.5[0.31-0.67] | 16% | 6% |
| ADAM.8 | 3.98 | 3.81 | 0.34 | 0.00 | 0.01 | 0.64[0.56-0.71] | 0.61[0.53-0.73] | 32% | 15% | 3.85 | 3.93 | -0.17 | 0.29 | 0.54 | 0.55[0.46-0.64] | 0.49[0.33-0.64] | 19% | 8% |
| ADAM.TS.15 | 3.51 | 3.52 | -0.03 | 0.79 | 0.86 | 0.51[0.43-0.59] | 0.46[0.37-0.55] | 17% | 6% | 3.54 | 3.53 | 0.04 | 0.55 | 0.73 | 0.53[0.44-0.62] | 0.49[0.38-0.63] | 18% | 8% |
| ALCAM | 4.83 | 4.87 | -0.08 | 0.04 | 0.12 | 0.58[0.5-0.66] | 0.53[0.35-0.67] | 23% | 12% | 4.85 | 4.91 | -0.12 | 0.20 | 0.42 | 0.56[0.47-0.65] | 0.51[0.34-0.66] | 19% | 10% |
| ANXA1 | 5.10 | 4.94 | 0.33 | 0.11 | 0.24 | 0.57[0.49-0.65] | 0.51[0.36-0.64] | 22% | 10% | 5.03 | 4.92 | 0.23 | 0.03 | 0.16 | 0.6[0.52-0.69] | 0.52[0.34-0.69] | 18% | 8% |
| AP.N | 4.72 | 4.87 | -0.32 | 0.01 | 0.03 | 0.61[0.53-0.69] | 0.55[0.38-0.7] | 29% | 16% | 4.84 | 4.86 | -0.04 | 0.83 | 0.89 | 0.51[0.41-0.61] | 0.46[0.35-0.58] | 17% | 7% |
| AREG | 1.27 | 0.76 | 1.01 | 0.00 | 0.00 | 0.79[0.72-0.85] | 0.77[0.69-0.87] | 62% | 52% | 1.25 | 0.90 | 0.70 | 0.00 | 0.00 | 0.72[0.64-0.8] | 0.7[0.61-0.83] | 53% | 35% |
| AREG.1 | 2.48 | 1.88 | 1.21 | 0.00 | 0.00 | 0.81[0.75-0.87] | 0.8[0.73-0.89] | 68% | 50% | 2.40 | 2.06 | 0.66 | 0.00 | 0.00 | 0.72[0.63-0.8] | 0.7[0.61-0.83] | 52% | 34% |
| ARNT | 0.71 | 0.71 | 0.00 | 0.16 | 0.32 | 0.46[0.4-0.52] | 0.47[0.4-0.58] | 17% | 8% | 0.71 | 0.71 | 0.00 | 0.75 | 0.86 | 0.49[0.43-0.55] | 0.48[0.42-0.58] | 16% | 8% |
| AXL | 7.94 | 8.04 | -0.20 | 0.44 | 0.60 | 0.53[0.45-0.61] | 0.47[0.36-0.58] | 17% | 7% | 8.08 | 8.02 | 0.13 | 0.98 | 0.98 | 0.5[0.41-0.6] | 0.45[0.36-0.56] | 15% | 7% |
| AZU1 | 4.73 | 4.54 | 0.38 | 0.06 | 0.14 | 0.58[0.5-0.66] | 0.53[0.41-0.67] | 22% | 12% | 4.81 | 4.56 | 0.50 | 0.40 | 0.63 | 0.54[0.45-0.63] | 0.46[0.35-0.59] | 11% | 6% |
| BACH1 | 1.52 | 1.41 | 0.22 | 0.68 | 0.78 | 0.52[0.44-0.6] | 0.46[0.36-0.57] | 16% | 6% | 1.60 | 1.22 | 0.76 | 0.03 | 0.16 | 0.6[0.51-0.69] | 0.51[0.31-0.69] | 19% | 6% |
| BIRC2 | 0.66 | 0.69 | -0.06 | 0.52 | 0.66 | 0.53[0.45-0.61] | 0.48[0.37-0.59] | 17% | 7% | 0.66 | 0.66 | 0.00 | 0.54 | 0.73 | 0.48[0.44-0.53] | 0.48[0.44-0.57] | 18% | 6% |
| BLM.hydrolase | 4.89 | 5.05 | -0.33 | 0.02 | 0.06 | 0.6[0.52-0.68] | 0.56[0.47-0.69] | 32% | 14% | 5.13 | 4.98 | 0.30 | 0.25 | 0.48 | 0.55[0.46-0.64] | 0.49[0.37-0.65] | 15% | 10% |
| BTN3A2 | 0.95 | 0.83 | 0.25 | 0.04 | 0.12 | 0.58[0.5-0.66] | 0.54[0.42-0.68] | 30% | 15% | 0.83 | 0.89 | -0.11 | 0.33 | 0.58 | 0.55[0.45-0.64] | 0.47[0.33-0.62] | 19% | 7% |
| CAIX | 3.37 | 2.98 | 0.78 | 0.00 | 0.00 | 0.64[0.57-0.72] | 0.63[0.54-0.74] | 45% | 35% | 3.10 | 3.12 | -0.04 | 0.67 | 0.82 | 0.48[0.38-0.58] | 0.49[0.37-0.63] | 21% | 13% |
| CASP.3 | 7.70 | 7.94 | -0.47 | 0.30 | 0.46 | 0.54[0.46-0.63] | 0.48[0.36-0.6] | 17% | 8% | 8.08 | 7.60 | 0.97 | 0.01 | 0.10 | 0.62[0.54-0.71] | 0.59[0.51-0.73] | 18% | 9% |
| CCL11 | 7.47 | 7.48 | -0.01 | 0.24 | 0.41 | 0.55[0.47-0.63] | 0.5[0.36-0.63] | 21% | 12% | 7.63 | 7.43 | 0.39 | 0.11 | 0.28 | 0.58[0.48-0.67] | 0.52[0.34-0.67] | 25% | 12% |
| CCL15 | 7.02 | 6.82 | 0.40 | 0.00 | 0.02 | 0.62[0.54-0.7] | 0.59[0.51-0.73] | 27% | 13% | 7.12 | 6.92 | 0.41 | 0.02 | 0.16 | 0.61[0.51-0.7] | 0.56[0.38-0.72] | 27% | 17% |
| CCL16 | 6.12 | 5.93 | 0.39 | 0.02 | 0.05 | 0.6[0.52-0.68] | 0.57[0.49-0.7] | 26% | 12% | 6.13 | 6.02 | 0.23 | 0.33 | 0.59 | 0.55[0.45-0.64] | 0.47[0.33-0.6] | 18% | 8% |
| CCL24 | 5.46 | 5.67 | -0.43 | 0.03 | 0.09 | 0.59[0.51-0.67] | 0.56[0.48-0.68] | 29% | 20% | 5.40 | 5.45 | -0.11 | 0.46 | 0.70 | 0.47[0.37-0.56] | 0.47[0.35-0.61] | 19% | 9% |
| CD160 | 5.15 | 5.18 | -0.06 | 0.60 | 0.73 | 0.52[0.44-0.6] | 0.46[0.36-0.57] | 17% | 9% | 5.07 | 5.19 | -0.24 | 0.05 | 0.18 | 0.59[0.5-0.68] | 0.53[0.34-0.69] | 23% | 12% |
| CD163 | 6.74 | 6.63 | 0.22 | 0.11 | 0.24 | 0.57[0.49-0.65] | 0.52[0.35-0.65] | 23% | 13% | 6.78 | 6.67 | 0.23 | 0.10 | 0.26 | 0.58[0.49-0.67] | 0.52[0.36-0.67] | 17% | 8% |
| CD207 | 2.68 | 2.58 | 0.20 | 0.08 | 0.19 | 0.57[0.49-0.65] | 0.54[0.46-0.67] | 29% | 16% | 2.58 | 2.63 | -0.10 | 0.66 | 0.82 | 0.52[0.43-0.61] | 0.46[0.35-0.6] | 16% | 8% |
| CD27 | 8.80 | 8.60 | 0.41 | 0.00 | 0.00 | 0.65[0.57-0.73] | 0.63[0.55-0.75] | 41% | 24% | 8.68 | 8.72 | -0.09 | 0.55 | 0.73 | 0.53[0.43-0.62] | 0.47[0.36-0.59] | 19% | 8% |
| CD28 | 0.70 | 0.69 | 0.02 | 0.36 | 0.52 | 0.54[0.46-0.62] | 0.49[0.39-0.61] | 20% | 10% | 0.69 | 0.69 | 0.00 | 0.86 | 0.90 | 0.5[0.45-0.54] | 0.46[0.44-0.53] | 14% | 6% |
| CD48 | 6.24 | 6.20 | 0.06 | 0.99 | 0.99 | 0.5[0.42-0.58] | 0.46[0.38-0.55] | 16% | 6% | 6.17 | 6.25 | -0.16 | 0.06 | 0.19 | 0.59[0.5-0.68] | 0.53[0.33-0.7] | 22% | 11% |
|  | **DISCOVERY SET** | | | | | | | | | **VALIDATION SET** | | | | | | | | |
| Marker | **CRC_**  **median** | **Control_**  **median** | **Fold Change** | **p_value** | **p_value^adj^** | **AUC^*^**  **(95% CI)** | **AUC^BS^**  **(95% CI)** | **Se^BS^ % at**  **80% Sp** | **Se^BS^ % at**  **90% Sp** | **CRC_**  **median** | **Control_**  **median** | **Fold Change** | **p_value** | **p_value^adj^** | **AUC^*^**  **(95% CI)** | **AUC^BS^**  **(95% CI)** | **Se^BS^ % at**  **80% Sp** | **Se^BS^ % at**  **90% Sp** |
| CD70 | 3.07 | 3.10 | -0.05 | 0.81 | 0.87 | 0.49[0.41-0.57] | 0.46[0.37-0.56] | 17% | 7% | 3.03 | 3.12 | -0.19 | 0.05 | 0.17 | 0.59[0.51-0.68] | 0.55[0.46-0.71] | 24% | 8% |
| CD83 | 2.10 | 2.11 | -0.02 | 0.81 | 0.87 | 0.49[0.41-0.57] | 0.46[0.37-0.56] | 17% | 6% | 2.06 | 2.19 | -0.27 | 0.09 | 0.24 | 0.58[0.49-0.68] | 0.51[0.32-0.67] | 22% | 10% |
| CD93 | 10.32 | 10.45 | -0.26 | 0.02 | 0.07 | 0.59[0.51-0.67] | 0.54[0.4-0.69] | 27% | 11% | 10.37 | 10.47 | -0.20 | 0.14 | 0.33 | 0.57[0.48-0.66] | 0.5[0.34-0.65] | 16% | 9% |
| CDH5 | 2.93 | 3.00 | -0.14 | 0.20 | 0.36 | 0.55[0.47-0.63] | 0.5[0.36-0.63] | 20% | 11% | 2.88 | 3.05 | -0.34 | 0.01 | 0.10 | 0.62[0.54-0.71] | 0.59[0.5-0.73] | 28% | 12% |
| CDSN | 2.89 | 2.86 | 0.07 | 0.33 | 0.48 | 0.54[0.46-0.62] | 0.5[0.37-0.62] | 19% | 11% | 2.78 | 2.96 | -0.35 | 0.35 | 0.61 | 0.54[0.45-0.64] | 0.47[0.35-0.61] | 17% | 8% |
| CEACAM1 | 6.75 | 6.79 | -0.07 | 0.36 | 0.52 | 0.54[0.46-0.62] | 0.48[0.36-0.6] | 18% | 9% | 6.75 | 6.80 | -0.11 | 0.39 | 0.63 | 0.54[0.45-0.63] | 0.47[0.34-0.61] | 15% | 8% |
| CEACAM5 | 2.08 | 1.05 | 2.05 | 0.00 | 0.00 | 0.73[0.66-0.8] | 0.72[0.65-0.82] | 54% | 44% | 1.96 | 1.36 | 1.20 | 0.00 | 0.00 | 0.72[0.63-0.81] | 0.71[0.6-0.84] | 60% | 45% |
| CHI3L1 | 6.69 | 6.16 | 1.06 | 0.01 | 0.03 | 0.61[0.53-0.69] | 0.58[0.49-0.7] | 31% | 17% | 6.69 | 6.21 | 0.96 | 0.04 | 0.17 | 0.6[0.5-0.69] | 0.55[0.43-0.7] | 27% | 15% |
| CHIT1 | 6.34 | 6.46 | -0.24 | 0.63 | 0.75 | 0.52[0.44-0.6] | 0.46[0.38-0.57] | 15% | 7% | 6.23 | 6.46 | -0.46 | 0.39 | 0.63 | 0.54[0.44-0.64] | 0.48[0.35-0.63] | 19% | 11% |
| CKAP4 | 4.24 | 4.02 | 0.45 | 0.00 | 0.00 | 0.65[0.57-0.72] | 0.62[0.53-0.75] | 41% | 27% | 4.31 | 4.10 | 0.43 | 0.03 | 0.16 | 0.6[0.51-0.7] | 0.55[0.38-0.72] | 26% | 15% |
| CLEC4A | 3.48 | 3.51 | -0.06 | 0.26 | 0.42 | 0.55[0.47-0.63] | 0.5[0.37-0.63] | 25% | 13% | 3.50 | 3.48 | 0.04 | 0.74 | 0.85 | 0.52[0.42-0.61] | 0.46[0.35-0.58] | 16% | 6% |
| CLEC4C | 2.32 | 2.39 | -0.13 | 0.79 | 0.86 | 0.49[0.41-0.57] | 0.46[0.39-0.57] | 16% | 9% | 2.28 | 2.40 | -0.24 | 0.16 | 0.37 | 0.57[0.47-0.66] | 0.5[0.32-0.65] | 22% | 10% |
| CLEC4D | 2.64 | 2.36 | 0.56 | 0.26 | 0.42 | 0.55[0.46-0.63] | 0.5[0.36-0.62] | 24% | 11% | 2.62 | 2.43 | 0.37 | 0.17 | 0.38 | 0.57[0.47-0.66] | 0.51[0.35-0.66] | 22% | 11% |
| CLEC4G | 2.74 | 2.73 | 0.02 | 0.53 | 0.66 | 0.53[0.44-0.61] | 0.47[0.37-0.58] | 15% | 8% | 2.75 | 2.73 | 0.03 | 0.70 | 0.83 | 0.52[0.42-0.62] | 0.44[0.34-0.57] | 15% | 7% |
| CLEC6A | 2.05 | 1.80 | 0.52 | 0.06 | 0.14 | 0.58[0.5-0.66] | 0.53[0.37-0.66] | 26% | 11% | 1.91 | 1.81 | 0.19 | 0.13 | 0.33 | 0.57[0.48-0.66] | 0.51[0.35-0.67] | 20% | 11% |
| CLEC7A | 3.10 | 3.03 | 0.16 | 0.27 | 0.43 | 0.55[0.46-0.63] | 0.49[0.37-0.62] | 17% | 10% | 3.07 | 3.03 | 0.09 | 0.67 | 0.82 | 0.52[0.43-0.61] | 0.45[0.35-0.57] | 14% | 6% |
| CNTN1 | 3.19 | 3.44 | -0.49 | 0.00 | 0.00 | 0.68[0.61-0.76] | 0.67[0.58-0.78] | 44% | 33% | 3.34 | 3.43 | -0.18 | 0.03 | 0.16 | 0.6[0.52-0.69] | 0.57[0.48-0.72] | 24% | 11% |
| CNTNAP2 | 1.21 | 1.17 | 0.10 | 0.51 | 0.65 | 0.53[0.45-0.61] | 0.47[0.36-0.59] | 17% | 8% | 1.17 | 1.23 | -0.11 | 0.37 | 0.63 | 0.54[0.45-0.63] | 0.48[0.35-0.62] | 18% | 8% |
| COL1A1 | 3.24 | 3.18 | 0.11 | 0.62 | 0.74 | 0.52[0.44-0.6] | 0.47[0.36-0.57] | 21% | 8% | 3.25 | 3.21 | 0.07 | 0.60 | 0.75 | 0.53[0.43-0.62] | 0.46[0.35-0.57] | 15% | 6% |
| CPA1 | 4.70 | 4.98 | -0.56 | 0.06 | 0.15 | 0.58[0.5-0.66] | 0.52[0.36-0.66] | 24% | 13% | 4.70 | 4.98 | -0.57 | 0.58 | 0.74 | 0.53[0.43-0.62] | 0.46[0.34-0.6] | 15% | 8% |
| CPB1 | 4.62 | 4.81 | -0.38 | 0.04 | 0.12 | 0.58[0.5-0.67] | 0.53[0.37-0.67] | 24% | 12% | 4.69 | 4.74 | -0.11 | 0.80 | 0.88 | 0.49[0.39-0.58] | 0.46[0.35-0.58] | 15% | 7% |
| CPE | 3.85 | 4.00 | -0.31 | 0.00 | 0.00 | 0.64[0.57-0.72] | 0.62[0.54-0.74] | 36% | 20% | 3.87 | 4.01 | -0.29 | 0.47 | 0.70 | 0.53[0.44-0.63] | 0.46[0.33-0.6] | 13% | 7% |
| CRNN | 4.70 | 4.77 | -0.15 | 0.19 | 0.35 | 0.55[0.47-0.64] | 0.5[0.35-0.63] | 20% | 12% | 4.61 | 4.73 | -0.25 | 0.21 | 0.42 | 0.56[0.47-0.65] | 0.49[0.35-0.66] | 19% | 10% |
| CSTB | 5.62 | 5.42 | 0.40 | 0.30 | 0.46 | 0.54[0.46-0.62] | 0.49[0.36-0.61] | 20% | 8% | 5.75 | 5.43 | 0.63 | 0.01 | 0.10 | 0.62[0.53-0.71] | 0.56[0.47-0.73] | 27% | 13% |
| CTSD | 4.20 | 4.12 | 0.17 | 0.08 | 0.20 | 0.57[0.49-0.65] | 0.54[0.46-0.67] | 30% | 19% | 4.26 | 4.07 | 0.37 | 0.01 | 0.09 | 0.63[0.54-0.72] | 0.6[0.51-0.74] | 34% | 18% |
| CTSV | 3.57 | 3.82 | -0.49 | 0.00 | 0.00 | 0.71[0.63-0.78] | 0.69[0.61-0.8] | 48% | 34% | 3.67 | 3.69 | -0.04 | 0.87 | 0.91 | 0.51[0.41-0.6] | 0.45[0.36-0.56] | 16% | 7% |
| CTSZ | 5.14 | 5.09 | 0.09 | 0.60 | 0.73 | 0.52[0.44-0.6] | 0.47[0.37-0.58] | 17% | 7% | 5.16 | 5.08 | 0.16 | 0.38 | 0.63 | 0.54[0.45-0.63] | 0.48[0.35-0.62] | 18% | 6% |
| CXADR | 1.42 | 1.37 | 0.10 | 0.63 | 0.75 | 0.52[0.44-0.6] | 0.48[0.38-0.6] | 21% | 11% | 1.43 | 1.36 | 0.14 | 0.23 | 0.45 | 0.56[0.47-0.65] | 0.49[0.33-0.64] | 19% | 10% |
|  | **DISCOVERY SET** | | | | | | | | | **VALIDATION SET** | | | | | | | | |
| Marker | **CRC_**  **median** | **Control_**  **median** | **Fold Change** | **p_value** | **p_value^adj^** | **AUC^*^**  **(95% CI)** | **AUC^BS^**  **(95% CI)** | **Se^BS^ % at**  **80% Sp** | **Se^BS^ % at**  **90% Sp** | **CRC_**  **median** | **Control_**  **median** | **Fold Change** | **p_value** | **p_value^adj^** | **AUC^*^**  **(95% CI)** | **AUC^BS^**  **(95% CI)** | **Se^BS^ % at**  **80% Sp** | **Se^BS^ % at**  **90% Sp** |
| CXCL12 | 1.14 | 1.13 | 0.02 | 0.31 | 0.47 | 0.54[0.46-0.62] | 0.5[0.39-0.63] | 20% | 11% | 1.18 | 1.09 | 0.17 | 0.04 | 0.17 | 0.6[0.51-0.69] | 0.55[0.44-0.7] | 20% | 9% |
| CXCL13 | 8.24 | 8.04 | 0.39 | 0.00 | 0.01 | 0.63[0.55-0.71] | 0.61[0.53-0.73] | 30% | 18% | 8.21 | 8.14 | 0.14 | 0.25 | 0.48 | 0.55[0.46-0.65] | 0.49[0.33-0.64] | 19% | 11% |
| CXCL16 | 4.93 | 4.92 | 0.02 | 0.68 | 0.78 | 0.48[0.4-0.57] | 0.46[0.37-0.56] | 16% | 9% | 4.91 | 4.95 | -0.08 | 0.70 | 0.83 | 0.52[0.43-0.61] | 0.46[0.35-0.58] | 18% | 7% |
| CXL17 | 4.70 | 4.54 | 0.32 | 0.09 | 0.20 | 0.57[0.49-0.65] | 0.5[0.34-0.65] | 20% | 7% | 4.69 | 4.61 | 0.16 | 0.07 | 0.21 | 0.59[0.5-0.67] | 0.54[0.46-0.69] | 23% | 11% |
| CYR61 | 5.50 | 5.70 | -0.40 | 0.04 | 0.10 | 0.59[0.51-0.67] | 0.54[0.37-0.68] | 30% | 16% | 5.70 | 5.56 | 0.27 | 0.29 | 0.53 | 0.55[0.46-0.64] | 0.49[0.34-0.63] | 21% | 8% |
| DAPP1 | 5.02 | 5.36 | -0.68 | 0.18 | 0.34 | 0.56[0.47-0.64] | 0.5[0.36-0.64] | 21% | 10% | 5.66 | 4.62 | 2.07 | 0.07 | 0.21 | 0.59[0.5-0.67] | 0.53[0.34-0.68] | 18% | 8% |
| DCBLD2 | 2.39 | 2.36 | 0.05 | 0.89 | 0.92 | 0.49[0.41-0.58] | 0.46[0.38-0.56] | 17% | 9% | 2.33 | 2.38 | -0.11 | 0.55 | 0.73 | 0.53[0.43-0.62] | 0.48[0.34-0.6] | 20% | 8% |
| DCTN1 | 4.25 | 4.66 | -0.81 | 0.24 | 0.41 | 0.55[0.47-0.63] | 0.48[0.35-0.62] | 16% | 6% | 4.13 | 4.26 | -0.25 | 0.84 | 0.89 | 0.51[0.42-0.6] | 0.46[0.36-0.58] | 12% | 6% |
| DDX58 | 2.51 | 2.46 | 0.12 | 0.16 | 0.32 | 0.56[0.48-0.64] | 0.5[0.35-0.63] | 18% | 10% | 2.75 | 2.35 | 0.79 | 0.07 | 0.21 | 0.59[0.49-0.68] | 0.5[0.31-0.67] | 20% | 9% |
| DFFA | 4.23 | 4.37 | -0.27 | 0.87 | 0.92 | 0.51[0.42-0.59] | 0.45[0.37-0.55] | 16% | 6% | 4.64 | 4.03 | 1.21 | 0.01 | 0.11 | 0.62[0.53-0.71] | 0.57[0.46-0.72] | 25% | 8% |
| DGKZ | 0.71 | 0.71 | 0.00 | 0.16 | 0.32 | 0.46[0.4-0.52] | 0.47[0.41-0.57] | 16% | 8% | 0.71 | 0.71 | 0.00 | 0.83 | 0.89 | 0.5[0.45-0.54] | 0.47[0.45-0.55] | 17% | 9% |
| DLK.1 | 5.41 | 5.56 | -0.30 | 0.01 | 0.03 | 0.61[0.53-0.69] | 0.58[0.5-0.71] | 33% | 19% | 5.32 | 5.65 | -0.65 | 0.00 | 0.08 | 0.63[0.55-0.72] | 0.6[0.52-0.74] | 27% | 14% |
| DLL1 | 9.44 | 9.33 | 0.23 | 0.00 | 0.00 | 0.67[0.59-0.74] | 0.65[0.56-0.77] | 35% | 26% | 9.36 | 9.41 | -0.11 | 0.47 | 0.70 | 0.53[0.44-0.63] | 0.46[0.34-0.59] | 15% | 7% |
| DPP10 | 0.57 | 0.59 | -0.05 | 0.64 | 0.75 | 0.48[0.4-0.56] | 0.48[0.38-0.59] | 18% | 10% | 0.54 | 0.58 | -0.08 | 0.74 | 0.85 | 0.52[0.42-0.61] | 0.45[0.36-0.57] | 17% | 7% |
| EDAR | 1.55 | 1.87 | -0.64 | 0.04 | 0.10 | 0.59[0.51-0.67] | 0.54[0.41-0.67] | 25% | 13% | 1.57 | 1.74 | -0.35 | 0.04 | 0.17 | 0.6[0.51-0.68] | 0.56[0.41-0.7] | 21% | 7% |
| EGF | 9.55 | 9.56 | -0.03 | 0.64 | 0.75 | 0.48[0.4-0.56] | 0.47[0.37-0.58] | 15% | 7% | 9.45 | 9.44 | 0.01 | 0.81 | 0.89 | 0.49[0.4-0.58] | 0.46[0.36-0.57] | 15% | 5% |
| EGFR | 2.57 | 2.75 | -0.36 | 0.00 | 0.00 | 0.73[0.66-0.8] | 0.72[0.63-0.82] | 49% | 34% | 2.66 | 2.65 | 0.01 | 0.39 | 0.63 | 0.46[0.37-0.55] | 0.47[0.34-0.62] | 15% | 9% |
| EGLN1 | 3.25 | 2.98 | 0.53 | 0.18 | 0.34 | 0.56[0.47-0.64] | 0.49[0.36-0.63] | 18% | 10% | 3.70 | 2.72 | 1.95 | 0.02 | 0.13 | 0.61[0.52-0.7] | 0.57[0.44-0.72] | 25% | 10% |
| EIF4G1 | 5.43 | 5.58 | -0.29 | 0.47 | 0.62 | 0.53[0.45-0.61] | 0.47[0.37-0.59] | 17% | 8% | 5.72 | 5.19 | 1.06 | 0.02 | 0.14 | 0.61[0.52-0.7] | 0.57[0.49-0.71] | 19% | 6% |
| EIF5A | 0.10 | 0.10 | 0.00 | 0.18 | 0.34 | 0.52[0.49-0.55] | 0.48[0.44-0.55] | 19% | 10% | 0.10 | 0.10 | 0.00 | 0.92 | 0.95 | 0.5[0.45-0.54] | 0.47[0.43-0.55] | 19% | 8% |
| Ep.CAM | 5.25 | 5.56 | -0.62 | 0.11 | 0.24 | 0.57[0.49-0.65] | 0.51[0.35-0.64] | 21% | 11% | 5.50 | 5.23 | 0.55 | 0.68 | 0.83 | 0.52[0.43-0.61] | 0.46[0.35-0.58] | 15% | 7% |
| EPHA2 | 2.55 | 2.41 | 0.29 | 0.00 | 0.01 | 0.64[0.56-0.72] | 0.62[0.53-0.73] | 35% | 22% | 2.48 | 2.50 | -0.03 | 0.45 | 0.70 | 0.54[0.44-0.63] | 0.46[0.34-0.58] | 14% | 9% |
| EPHB4 | 4.22 | 4.08 | 0.27 | 0.00 | 0.00 | 0.64[0.56-0.72] | 0.62[0.53-0.74] | 35% | 19% | 4.23 | 4.17 | 0.13 | 0.77 | 0.87 | 0.49[0.39-0.58] | 0.45[0.36-0.56] | 15% | 6% |
| ERBB2 | 7.42 | 7.61 | -0.37 | 0.00 | 0.00 | 0.66[0.58-0.73] | 0.64[0.56-0.76] | 43% | 23% | 7.51 | 7.58 | -0.13 | 0.27 | 0.51 | 0.55[0.46-0.64] | 0.49[0.33-0.64] | 19% | 10% |
| ERBB3 | 7.69 | 7.80 | -0.22 | 0.00 | 0.00 | 0.65[0.57-0.73] | 0.63[0.55-0.75] | 39% | 23% | 7.72 | 7.73 | -0.01 | 0.53 | 0.73 | 0.53[0.44-0.62] | 0.48[0.35-0.61] | 20% | 7% |
| ERBB4 | 4.82 | 4.95 | -0.27 | 0.00 | 0.00 | 0.65[0.57-0.73] | 0.63[0.54-0.75] | 44% | 30% | 4.87 | 4.96 | -0.19 | 0.06 | 0.19 | 0.59[0.5-0.68] | 0.53[0.31-0.69] | 22% | 9% |
| ESM.1 | 8.98 | 8.87 | 0.22 | 0.00 | 0.01 | 0.63[0.55-0.71] | 0.61[0.53-0.73] | 34% | 21% | 8.94 | 9.03 | -0.18 | 0.97 | 0.98 | 0.5[0.41-0.59] | 0.46[0.36-0.57] | 17% | 6% |
| FABP4 | 5.26 | 4.93 | 0.66 | 0.01 | 0.05 | 0.6[0.52-0.68] | 0.57[0.48-0.7] | 30% | 16% | 5.17 | 5.10 | 0.13 | 0.80 | 0.88 | 0.51[0.42-0.6] | 0.46[0.36-0.57] | 14% | 6% |
|  | **DISCOVERY SET** | | | | | | | | | **VALIDATION SET** | | | | | | | | |
| Marker | **CRC_**  **median** | **Control_**  **median** | **Fold Change** | **p_value** | **p_value^adj^** | **AUC^*^**  **(95% CI)** | **AUC^BS^**  **(95% CI)** | **Se^BS^ % at**  **80% Sp** | **Se^BS^ % at**  **90% Sp** | **CRC_**  **median** | **Control_**  **median** | **Fold Change** | **p_value** | **p_value^adj^** | **AUC^*^**  **(95% CI)** | **AUC^BS^**  **(95% CI)** | **Se^BS^ % at**  **80% Sp** | **Se^BS^ % at**  **90% Sp** |
| FADD | 2.32 | 2.30 | 0.04 | 0.46 | 0.62 | 0.53[0.45-0.61] | 0.48[0.36-0.6] | 20% | 7% | 2.55 | 2.21 | 0.68 | 0.15 | 0.36 | 0.57[0.48-0.66] | 0.49[0.31-0.65] | 16% | 6% |
| FAM3B | 3.92 | 3.99 | -0.14 | 0.14 | 0.28 | 0.56[0.48-0.64] | 0.52[0.37-0.65] | 20% | 12% | 4.02 | 3.93 | 0.18 | 0.77 | 0.87 | 0.49[0.39-0.58] | 0.46[0.35-0.6] | 21% | 9% |
| FAS | 4.79 | 4.80 | -0.02 | 0.38 | 0.53 | 0.54[0.46-0.62] | 0.49[0.37-0.62] | 17% | 8% | 4.86 | 4.80 | 0.12 | 0.71 | 0.84 | 0.52[0.42-0.61] | 0.46[0.35-0.58] | 15% | 8% |
| FASLG | 8.89 | 9.13 | -0.48 | 0.00 | 0.00 | 0.65[0.58-0.73] | 0.63[0.55-0.75] | 32% | 18% | 8.99 | 8.97 | 0.02 | 0.96 | 0.98 | 0.5[0.4-0.59] | 0.46[0.35-0.57] | 15% | 6% |
| FCRL3 | 0.18 | 0.27 | -0.18 | 0.19 | 0.35 | 0.55[0.47-0.64] | 0.48[0.34-0.61] | 18% | 10% | 0.25 | 0.26 | -0.02 | 0.91 | 0.95 | 0.49[0.4-0.59] | 0.45[0.36-0.56] | 15% | 6% |
| FCRL6 | 1.71 | 1.56 | 0.29 | 0.17 | 0.32 | 0.56[0.48-0.64] | 0.5[0.35-0.63] | 18% | 10% | 1.62 | 1.77 | -0.30 | 0.54 | 0.73 | 0.53[0.44-0.62] | 0.47[0.35-0.58] | 18% | 7% |
| FCRLB | 1.36 | 1.30 | 0.13 | 0.44 | 0.60 | 0.53[0.45-0.61] | 0.48[0.37-0.6] | 18% | 10% | 1.36 | 1.33 | 0.07 | 0.52 | 0.73 | 0.53[0.44-0.62] | 0.47[0.34-0.61] | 18% | 9% |
| FGF.BP1 | 5.02 | 5.06 | -0.08 | 0.79 | 0.86 | 0.51[0.43-0.59] | 0.47[0.37-0.59] | 19% | 13% | 4.99 | 5.02 | -0.06 | 0.79 | 0.88 | 0.49[0.39-0.58] | 0.48[0.36-0.62] | 21% | 12% |
| FGF2 | 0.69 | 0.81 | -0.26 | 0.62 | 0.74 | 0.52[0.44-0.6] | 0.46[0.37-0.56] | 16% | 9% | 0.70 | 0.71 | -0.01 | 0.63 | 0.79 | 0.48[0.39-0.57] | 0.45[0.34-0.56] | 15% | 6% |
| FR.alpha | 6.35 | 6.35 | 0.00 | 0.87 | 0.92 | 0.49[0.41-0.57] | 0.46[0.38-0.56] | 16% | 8% | 6.25 | 6.47 | -0.43 | 0.02 | 0.12 | 0.61[0.52-0.71] | 0.55[0.37-0.72] | 29% | 18% |
| FR.gamma | 6.42 | 6.36 | 0.13 | 0.21 | 0.37 | 0.55[0.47-0.63] | 0.46[0.36-0.57] | 15% | 8% | 6.36 | 6.43 | -0.14 | 0.20 | 0.42 | 0.56[0.47-0.65] | 0.47[0.35-0.6] | 15% | 6% |
| FURIN | 8.89 | 8.72 | 0.33 | 0.00 | 0.01 | 0.63[0.55-0.71] | 0.6[0.51-0.72] | 29% | 14% | 8.84 | 8.79 | 0.09 | 0.35 | 0.61 | 0.54[0.45-0.64] | 0.48[0.34-0.62] | 18% | 6% |
| FXYD5 | 0.28 | 0.41 | -0.26 | 0.13 | 0.27 | 0.56[0.48-0.64] | 0.49[0.35-0.63] | 19% | 8% | 0.34 | 0.34 | 0.00 | 0.63 | 0.79 | 0.52[0.44-0.6] | 0.45[0.36-0.55] | 15% | 8% |
| Gal.1 | 6.52 | 6.54 | -0.04 | 0.52 | 0.66 | 0.47[0.39-0.55] | 0.48[0.37-0.6] | 18% | 7% | 6.47 | 6.57 | -0.20 | 0.01 | 0.10 | 0.62[0.53-0.71] | 0.59[0.49-0.74] | 26% | 13% |
| Gal.3 | 5.72 | 5.67 | 0.08 | 0.84 | 0.89 | 0.51[0.43-0.59] | 0.46[0.38-0.55] | 16% | 6% | 5.77 | 5.66 | 0.22 | 0.13 | 0.31 | 0.57[0.48-0.67] | 0.51[0.33-0.67] | 20% | 11% |
| Gal.4 | 2.85 | 2.54 | 0.63 | 0.00 | 0.00 | 0.67[0.59-0.75] | 0.65[0.57-0.77] | 41% | 30% | 2.97 | 2.69 | 0.55 | 0.01 | 0.11 | 0.62[0.53-0.71] | 0.57[0.48-0.73] | 28% | 15% |
| GALNT3 | 1.03 | 1.05 | -0.02 | 0.52 | 0.66 | 0.47[0.39-0.56] | 0.48[0.36-0.6] | 18% | 11% | 1.04 | 1.04 | 0.00 | 0.76 | 0.87 | 0.51[0.44-0.59] | 0.46[0.39-0.57] | 15% | 9% |
| GDF.15 | 5.56 | 4.92 | 1.27 | 0.00 | 0.00 | 0.76[0.69-0.82] | 0.75[0.66-0.84] | 60% | 38% | 5.35 | 5.05 | 0.60 | 0.00 | 0.08 | 0.63[0.54-0.73] | 0.6[0.51-0.75] | 32% | 19% |
| GLB1 | -0.41 | -0.34 | -0.15 | 0.12 | 0.26 | 0.56[0.48-0.65] | 0.52[0.39-0.65] | 18% | 9% | -0.43 | -0.34 | -0.17 | 0.03 | 0.16 | 0.6[0.51-0.69] | 0.56[0.49-0.71] | 20% | 11% |
| GPC1 | 4.44 | 4.54 | -0.19 | 0.05 | 0.13 | 0.58[0.5-0.66] | 0.54[0.47-0.67] | 27% | 16% | 4.45 | 4.58 | -0.27 | 0.07 | 0.21 | 0.59[0.49-0.68] | 0.54[0.38-0.69] | 25% | 16% |
| GPNMB | 6.50 | 6.55 | -0.10 | 0.22 | 0.39 | 0.55[0.47-0.63] | 0.47[0.35-0.6] | 20% | 7% | 6.51 | 6.52 | -0.02 | 0.98 | 0.98 | 0.5[0.41-0.59] | 0.45[0.37-0.56] | 14% | 5% |
| GRN | 6.13 | 6.07 | 0.12 | 0.16 | 0.32 | 0.56[0.48-0.64] | 0.5[0.35-0.63] | 24% | 10% | 6.21 | 6.14 | 0.14 | 0.14 | 0.34 | 0.57[0.48-0.66] | 0.5[0.32-0.66] | 22% | 12% |
| GZMB | 4.42 | 5.38 | -1.92 | 0.00 | 0.00 | 0.65[0.57-0.72] | 0.63[0.54-0.75] | 36% | 12% | 5.11 | 4.79 | 0.64 | 0.16 | 0.37 | 0.57[0.48-0.66] | 0.5[0.36-0.66] | 19% | 10% |
| GZMH | 4.51 | 5.10 | -1.18 | 0.02 | 0.05 | 0.6[0.52-0.68] | 0.58[0.48-0.7] | 23% | 10% | 4.97 | 4.67 | 0.60 | 0.05 | 0.17 | 0.59[0.51-0.68] | 0.53[0.38-0.71] | 21% | 12% |
| HCLS1 | 6.32 | 6.37 | -0.11 | 0.76 | 0.85 | 0.49[0.41-0.57] | 0.47[0.38-0.58] | 15% | 8% | 6.62 | 6.13 | 0.97 | 0.03 | 0.16 | 0.6[0.51-0.69] | 0.56[0.47-0.71] | 21% | 10% |
| HEXIM1 | 4.25 | 4.25 | -0.02 | 0.67 | 0.78 | 0.52[0.44-0.6] | 0.46[0.37-0.56] | 16% | 6% | 4.62 | 3.95 | 1.35 | 0.01 | 0.10 | 0.62[0.54-0.71] | 0.58[0.5-0.74] | 25% | 9% |
| HGF | 8.05 | 7.61 | 0.89 | 0.00 | 0.00 | 0.67[0.6-0.75] | 0.66[0.57-0.77] | 43% | 24% | 7.86 | 7.72 | 0.29 | 0.07 | 0.21 | 0.59[0.49-0.68] | 0.54[0.42-0.69] | 22% | 14% |
| hK11 | 6.01 | 5.91 | 0.19 | 0.21 | 0.36 | 0.55[0.47-0.63] | 0.51[0.36-0.64] | 19% | 12% | 5.80 | 5.99 | -0.39 | 0.03 | 0.16 | 0.61[0.51-0.7] | 0.54[0.33-0.7] | 29% | 15% |
|  | **DISCOVERY SET** | | | | | | | | | **VALIDATION SET** | | | | | | | | |
| Marker | **CRC_**  **median** | **Control_**  **median** | **Fold Change** | **p_value** | **p_value^adj^** | **AUC^*^**  **(95% CI)** | **AUC^BS^**  **(95% CI)** | **Se^BS^ % at**  **80% Sp** | **Se^BS^ % at**  **90% Sp** | **CRC_**  **median** | **Control_**  **median** | **Fold Change** | **p_value** | **p_value^adj^** | **AUC^*^**  **(95% CI)** | **AUC^BS^**  **(95% CI)** | **Se^BS^ % at**  **80% Sp** | **Se^BS^ % at**  **90% Sp** |
| hK14 | 6.71 | 6.73 | -0.05 | 0.84 | 0.90 | 0.51[0.43-0.59] | 0.46[0.37-0.56] | 16% | 9% | 6.71 | 6.68 | 0.06 | 0.71 | 0.84 | 0.52[0.42-0.61] | 0.46[0.36-0.58] | 16% | 6% |
| hK8 | 6.70 | 6.67 | 0.05 | 0.67 | 0.78 | 0.48[0.4-0.56] | 0.47[0.38-0.58] | 17% | 9% | 6.60 | 6.80 | -0.40 | 0.02 | 0.12 | 0.61[0.52-0.71] | 0.58[0.48-0.73] | 28% | 15% |
| HNMT | 0.40 | 0.35 | 0.08 | 0.46 | 0.61 | 0.53[0.45-0.61] | 0.49[0.39-0.61] | 22% | 11% | 0.34 | 0.39 | -0.10 | 0.77 | 0.87 | 0.49[0.39-0.58] | 0.46[0.36-0.59] | 17% | 6% |
| HSD11B1 | 2.11 | 2.27 | -0.32 | 0.01 | 0.02 | 0.62[0.54-0.69] | 0.58[0.5-0.7] | 24% | 9% | 2.19 | 2.27 | -0.17 | 0.51 | 0.73 | 0.53[0.44-0.62] | 0.47[0.34-0.6] | 18% | 7% |
| ICA1 | 1.65 | 1.76 | -0.23 | 0.34 | 0.50 | 0.54[0.46-0.62] | 0.48[0.36-0.6] | 16% | 8% | 1.80 | 1.74 | 0.13 | 0.20 | 0.42 | 0.56[0.47-0.64] | 0.45[0.33-0.56] | 16% | 6% |
| ICAM.2 | 4.39 | 4.31 | 0.15 | 0.06 | 0.14 | 0.58[0.5-0.66] | 0.54[0.45-0.68] | 27% | 16% | 4.36 | 4.42 | -0.12 | 0.45 | 0.70 | 0.54[0.44-0.63] | 0.47[0.34-0.6] | 18% | 7% |
| ICOSLG | 4.80 | 4.90 | -0.19 | 0.00 | 0.02 | 0.62[0.54-0.7] | 0.6[0.51-0.72] | 34% | 23% | 4.79 | 4.91 | -0.25 | 0.01 | 0.10 | 0.62[0.53-0.71] | 0.59[0.5-0.73] | 29% | 15% |
| IFN.gamma.R1 | 4.52 | 4.41 | 0.22 | 0.02 | 0.06 | 0.6[0.52-0.68] | 0.56[0.48-0.69] | 30% | 13% | 4.41 | 4.49 | -0.17 | 0.04 | 0.17 | 0.6[0.5-0.69] | 0.54[0.34-0.7] | 25% | 13% |
| IFNLR1 | 1.83 | 1.87 | -0.09 | 0.23 | 0.39 | 0.55[0.47-0.63] | 0.51[0.37-0.65] | 22% | 10% | 1.78 | 1.89 | -0.22 | 0.25 | 0.48 | 0.55[0.46-0.65] | 0.49[0.34-0.64] | 17% | 10% |
| IGF1R | 3.12 | 3.14 | -0.04 | 0.81 | 0.87 | 0.51[0.43-0.59] | 0.46[0.37-0.55] | 17% | 9% | 3.09 | 3.17 | -0.17 | 0.05 | 0.17 | 0.59[0.5-0.69] | 0.54[0.35-0.7] | 25% | 13% |
| IGFBP.1 | 4.60 | 4.65 | -0.11 | 0.97 | 0.98 | 0.5[0.42-0.58] | 0.46[0.38-0.56] | 17% | 7% | 4.75 | 4.69 | 0.11 | 0.37 | 0.63 | 0.54[0.45-0.64] | 0.48[0.34-0.63] | 24% | 10% |
| IGFBP.2 | 8.18 | 7.86 | 0.65 | 0.00 | 0.02 | 0.62[0.54-0.7] | 0.59[0.51-0.72] | 34% | 22% | 8.11 | 7.97 | 0.29 | 0.43 | 0.66 | 0.54[0.44-0.63] | 0.48[0.34-0.62] | 22% | 10% |
| IGFBP.7 | 6.51 | 6.49 | 0.03 | 0.61 | 0.73 | 0.52[0.44-0.6] | 0.49[0.38-0.6] | 22% | 11% | 6.53 | 6.51 | 0.04 | 0.71 | 0.84 | 0.48[0.39-0.58] | 0.46[0.36-0.59] | 15% | 8% |
| IL.17RA | 3.55 | 3.63 | -0.14 | 0.95 | 0.96 | 0.5[0.42-0.58] | 0.47[0.38-0.57] | 19% | 9% | 3.58 | 3.57 | 0.00 | 0.81 | 0.89 | 0.51[0.42-0.6] | 0.46[0.36-0.56] | 15% | 5% |
| IL.18BP | 5.72 | 5.57 | 0.30 | 0.01 | 0.03 | 0.61[0.53-0.69] | 0.59[0.5-0.71] | 33% | 21% | 5.71 | 5.70 | 0.01 | 0.86 | 0.91 | 0.51[0.42-0.6] | 0.45[0.36-0.58] | 16% | 6% |
| IL.1RT1 | 5.96 | 5.86 | 0.21 | 0.00 | 0.01 | 0.62[0.54-0.7] | 0.6[0.52-0.73] | 37% | 27% | 6.01 | 5.90 | 0.23 | 0.12 | 0.31 | 0.57[0.48-0.66] | 0.52[0.36-0.67] | 22% | 9% |
| IL.1RT2 | 5.11 | 5.23 | -0.25 | 0.00 | 0.00 | 0.64[0.57-0.72] | 0.63[0.54-0.75] | 37% | 19% | 5.20 | 5.20 | 0.00 | 0.82 | 0.89 | 0.51[0.42-0.6] | 0.46[0.35-0.57] | 16% | 7% |
| IL.6RA | 11.70 | 11.73 | -0.05 | 0.77 | 0.85 | 0.51[0.43-0.59] | 0.47[0.38-0.58] | 17% | 8% | 11.74 | 11.73 | 0.01 | 0.64 | 0.79 | 0.52[0.43-0.62] | 0.47[0.35-0.6] | 17% | 8% |
| IL10 | 1.68 | 1.58 | 0.20 | 0.12 | 0.26 | 0.56[0.48-0.65] | 0.49[0.33-0.64] | 22% | 9% | 1.56 | 1.67 | -0.22 | 0.04 | 0.17 | 0.6[0.5-0.69] | 0.54[0.42-0.7] | 25% | 14% |
| IL12RB1 | 1.46 | 1.35 | 0.23 | 0.09 | 0.20 | 0.57[0.49-0.65] | 0.51[0.35-0.65] | 22% | 13% | 1.40 | 1.38 | 0.04 | 0.24 | 0.47 | 0.56[0.46-0.65] | 0.49[0.33-0.64] | 17% | 10% |
| IL2.RA | 3.91 | 3.67 | 0.47 | 0.00 | 0.00 | 0.64[0.57-0.72] | 0.63[0.54-0.74] | 39% | 24% | 3.92 | 3.72 | 0.41 | 0.00 | 0.08 | 0.64[0.55-0.72] | 0.59[0.51-0.74] | 30% | 11% |
| IL5 | 0.84 | 0.84 | 0.00 | 0.32 | 0.47 | 0.46[0.39-0.54] | 0.49[0.39-0.61] | 16% | 8% | 0.84 | 0.84 | 0.00 | 0.09 | 0.25 | 0.56[0.49-0.63] | 0.46[0.37-0.58] | 17% | 7% |
| IL6 | 2.37 | 1.59 | 1.57 | 0.00 | 0.00 | 0.75[0.68-0.82] | 0.74[0.65-0.84] | 56% | 31% | 1.96 | 1.85 | 0.22 | 0.04 | 0.17 | 0.6[0.51-0.69] | 0.54[0.4-0.7] | 25% | 13% |
| IL6.1 | 2.90 | 2.06 | 1.68 | 0.00 | 0.00 | 0.76[0.69-0.83] | 0.75[0.67-0.85] | 54% | 34% | 2.56 | 2.38 | 0.35 | 0.03 | 0.16 | 0.61[0.52-0.69] | 0.56[0.48-0.71] | 26% | 13% |
| IRAK1 | 1.19 | 1.37 | -0.35 | 0.25 | 0.42 | 0.55[0.47-0.63] | 0.48[0.35-0.61] | 16% | 8% | 1.29 | 1.22 | 0.15 | 0.49 | 0.71 | 0.53[0.44-0.62] | 0.46[0.35-0.58] | 15% | 5% |
| IRAK4 | 3.66 | 3.90 | -0.48 | 0.36 | 0.51 | 0.54[0.46-0.62] | 0.48[0.36-0.59] | 17% | 8% | 4.12 | 3.43 | 1.37 | 0.10 | 0.26 | 0.58[0.49-0.67] | 0.52[0.33-0.67] | 20% | 6% |
| IRF9 | 1.66 | 1.69 | -0.06 | 0.27 | 0.43 | 0.45[0.37-0.54] | 0.48[0.35-0.61] | 19% | 6% | 2.07 | 1.52 | 1.09 | 0.02 | 0.13 | 0.61[0.52-0.7] | 0.51[0.29-0.69] | 18% | 7% |
| ITGA11 | 1.98 | 2.53 | -1.10 | 0.00 | 0.00 | 0.8[0.73-0.86] | 0.78[0.71-0.87] | 60% | 48% | 2.27 | 2.28 | -0.03 | 0.20 | 0.42 | 0.56[0.47-0.65] | 0.51[0.39-0.66] | 17% | 8% |
|  | **DISCOVERY SET** | | | | | | | | | **VALIDATION SET** | | | | | | | | |
| Marker | **CRC_**  **median** | **Control_**  **median** | **Fold Change** | **p_value** | **p_value^adj^** | **AUC^*^**  **(95% CI)** | **AUC^BS^**  **(95% CI)** | **Se^BS^ % at**  **80% Sp** | **Se^BS^ % at**  **90% Sp** | **CRC_**  **median** | **Control_**  **median** | **Fold Change** | **p_value** | **p_value^adj^** | **AUC^*^**  **(95% CI)** | **AUC^BS^**  **(95% CI)** | **Se^BS^ % at**  **80% Sp** | **Se^BS^ % at**  **90% Sp** |
| ITGA6 | 0.84 | 1.03 | -0.36 | 0.14 | 0.28 | 0.56[0.48-0.64] | 0.5[0.35-0.64] | 17% | 7% | 1.00 | 0.78 | 0.45 | 0.17 | 0.37 | 0.57[0.47-0.66] | 0.49[0.33-0.64] | 16% | 6% |
| ITGAV | 3.54 | 3.78 | -0.49 | 0.00 | 0.00 | 0.78[0.71-0.84] | 0.77[0.69-0.85] | 61% | 50% | 3.63 | 3.75 | -0.24 | 0.01 | 0.10 | 0.62[0.54-0.71] | 0.59[0.5-0.74] | 30% | 16% |
| ITGB2 | 5.82 | 6.01 | -0.37 | 0.01 | 0.03 | 0.61[0.53-0.69] | 0.58[0.5-0.71] | 29% | 18% | 6.04 | 5.83 | 0.41 | 0.06 | 0.19 | 0.59[0.5-0.68] | 0.53[0.41-0.69] | 20% | 9% |
| ITGB5 | 8.01 | 8.08 | -0.14 | 0.30 | 0.46 | 0.54[0.46-0.63] | 0.47[0.35-0.6] | 20% | 10% | 8.08 | 8.01 | 0.13 | 0.52 | 0.73 | 0.53[0.44-0.62] | 0.47[0.35-0.6] | 14% | 7% |
| ITGB6 | 1.54 | 1.85 | -0.61 | 0.00 | 0.00 | 0.68[0.61-0.76] | 0.67[0.58-0.78] | 43% | 24% | 1.66 | 1.71 | -0.09 | 0.10 | 0.26 | 0.58[0.49-0.67] | 0.54[0.43-0.69] | 23% | 13% |
| ITM2A | 1.74 | 1.63 | 0.21 | 0.07 | 0.17 | 0.58[0.49-0.66] | 0.53[0.41-0.66] | 22% | 12% | 1.76 | 1.67 | 0.19 | 0.73 | 0.85 | 0.52[0.42-0.61] | 0.46[0.35-0.58] | 17% | 7% |
| JAM.A | 5.94 | 5.91 | 0.04 | 0.42 | 0.58 | 0.47[0.38-0.55] | 0.48[0.37-0.6] | 19% | 8% | 6.03 | 5.66 | 0.75 | 0.04 | 0.17 | 0.6[0.51-0.69] | 0.54[0.39-0.71] | 22% | 11% |
| JUN | 0.35 | 0.35 | 0.00 | 0.05 | 0.13 | 0.44[0.39-0.5] | 0.47[0.38-0.58] | 15% | 7% | 0.35 | 0.35 | 0.00 | 0.40 | 0.63 | 0.53[0.46-0.59] | 0.46[0.4-0.57] | 15% | 9% |
| KLK13 | 3.38 | 3.50 | -0.25 | 0.17 | 0.33 | 0.56[0.48-0.64] | 0.51[0.42-0.64] | 22% | 13% | 3.43 | 3.42 | 0.03 | 0.91 | 0.95 | 0.51[0.41-0.6] | 0.45[0.36-0.58] | 17% | 7% |
| KLK6 | 4.76 | 4.80 | -0.07 | 0.67 | 0.78 | 0.48[0.4-0.56] | 0.47[0.38-0.58] | 17% | 9% | 4.80 | 4.86 | -0.13 | 0.97 | 0.98 | 0.5[0.41-0.59] | 0.46[0.36-0.59] | 16% | 9% |
| KLRD1 | 5.06 | 5.05 | 0.01 | 0.93 | 0.95 | 0.5[0.41-0.58] | 0.46[0.38-0.55] | 16% | 6% | 5.11 | 5.10 | 0.02 | 0.92 | 0.95 | 0.5[0.4-0.59] | 0.45[0.35-0.56] | 16% | 7% |
| KPNA1 | 0.78 | 0.78 | 0.00 | 0.53 | 0.66 | 0.51[0.47-0.55] | 0.48[0.44-0.56] | 18% | 9% | 0.78 | 0.78 | 0.00 | 0.50 | 0.72 | 0.49[0.45-0.52] | 0.48[0.46-0.56] | 15% | 8% |
| KRT19 | 2.99 | 1.99 | 2.00 | 0.00 | 0.00 | 0.76[0.69-0.83] | 0.75[0.67-0.85] | 55% | 43% | 2.88 | 2.27 | 1.23 | 0.00 | 0.00 | 0.72[0.63-0.8] | 0.7[0.6-0.83] | 51% | 37% |
| LAG3 | 1.46 | 1.43 | 0.07 | 0.45 | 0.61 | 0.53[0.45-0.61] | 0.49[0.38-0.61] | 20% | 10% | 1.40 | 1.55 | -0.31 | 0.03 | 0.16 | 0.6[0.51-0.69] | 0.54[0.38-0.7] | 25% | 13% |
| LAMP3 | 2.85 | 2.93 | -0.18 | 0.74 | 0.84 | 0.51[0.43-0.6] | 0.46[0.37-0.55] | 16% | 9% | 2.91 | 2.88 | 0.05 | 0.78 | 0.88 | 0.49[0.39-0.58] | 0.45[0.35-0.57] | 15% | 7% |
| LDL.receptor | 4.24 | 4.42 | -0.36 | 0.24 | 0.41 | 0.55[0.47-0.63] | 0.5[0.36-0.64] | 22% | 13% | 4.31 | 4.28 | 0.06 | 0.71 | 0.84 | 0.52[0.42-0.61] | 0.45[0.35-0.56] | 15% | 6% |
| LILRB4 | 2.71 | 2.45 | 0.53 | 0.00 | 0.02 | 0.62[0.54-0.7] | 0.59[0.51-0.71] | 34% | 26% | 2.56 | 2.55 | 0.01 | 0.18 | 0.38 | 0.56[0.47-0.66] | 0.52[0.37-0.67] | 24% | 13% |
| LTBR | 3.64 | 3.51 | 0.27 | 0.00 | 0.01 | 0.64[0.56-0.72] | 0.62[0.54-0.74] | 38% | 22% | 3.57 | 3.60 | -0.07 | 0.72 | 0.84 | 0.48[0.39-0.58] | 0.47[0.35-0.6] | 22% | 9% |
| LY75 | 1.77 | 1.77 | 0.00 | 0.25 | 0.41 | 0.46[0.39-0.53] | 0.46[0.37-0.55] | 20% | 13% | 1.73 | 1.77 | -0.07 | 0.59 | 0.75 | 0.53[0.43-0.62] | 0.46[0.35-0.58] | 18% | 8% |
| LY9 | 5.18 | 5.14 | 0.09 | 0.77 | 0.85 | 0.49[0.41-0.57] | 0.46[0.37-0.57] | 16% | 8% | 4.98 | 5.20 | -0.43 | 0.02 | 0.16 | 0.61[0.51-0.7] | 0.53[0.29-0.7] | 23% | 10% |
| LYN | 3.38 | 3.40 | -0.04 | 0.69 | 0.78 | 0.52[0.44-0.6] | 0.46[0.37-0.55] | 16% | 6% | 3.75 | 3.14 | 1.23 | 0.01 | 0.10 | 0.62[0.53-0.71] | 0.59[0.5-0.73] | 21% | 10% |
| LYPD3 | 3.88 | 4.07 | -0.39 | 0.00 | 0.00 | 0.65[0.57-0.73] | 0.63[0.55-0.76] | 38% | 21% | 4.01 | 4.01 | 0.00 | 0.58 | 0.74 | 0.53[0.43-0.62] | 0.48[0.35-0.63] | 22% | 11% |
| MAD.homolog.5 | 3.87 | 3.89 | -0.04 | 0.56 | 0.70 | 0.52[0.44-0.61] | 0.49[0.38-0.61] | 20% | 13% | 3.90 | 3.88 | 0.03 | 0.58 | 0.74 | 0.53[0.43-0.62] | 0.46[0.34-0.59] | 16% | 7% |
| MASP1 | 1.04 | 1.23 | -0.38 | 0.00 | 0.00 | 0.65[0.57-0.73] | 0.63[0.55-0.75] | 33% | 22% | 1.07 | 1.17 | -0.20 | 0.08 | 0.23 | 0.58[0.49-0.68] | 0.53[0.37-0.68] | 25% | 13% |
| MB | 6.37 | 6.55 | -0.36 | 0.01 | 0.05 | 0.6[0.52-0.68] | 0.57[0.49-0.71] | 29% | 17% | 6.47 | 6.59 | -0.23 | 0.20 | 0.42 | 0.56[0.46-0.66] | 0.49[0.32-0.62] | 22% | 11% |
| MCP.1 | 3.52 | 3.85 | -0.64 | 0.00 | 0.00 | 0.66[0.59-0.74] | 0.64[0.56-0.76] | 44% | 26% | 3.78 | 3.65 | 0.27 | 0.48 | 0.70 | 0.53[0.44-0.63] | 0.48[0.34-0.63] | 18% | 8% |
| MEPE | 4.14 | 4.21 | -0.14 | 0.55 | 0.68 | 0.53[0.44-0.61] | 0.47[0.37-0.57] | 19% | 7% | 4.22 | 4.19 | 0.07 | 0.57 | 0.74 | 0.53[0.43-0.62] | 0.47[0.35-0.59] | 16% | 8% |
| MetAP.2 | 5.07 | 5.06 | 0.02 | 0.85 | 0.91 | 0.49[0.41-0.57] | 0.46[0.38-0.55] | 16% | 6% | 5.23 | 4.91 | 0.63 | 0.04 | 0.17 | 0.6[0.51-0.69] | 0.55[0.38-0.7] | 19% | 10% |
|  | **DISCOVERY SET** | | | | | | | | | **VALIDATION SET** | | | | | | | | |
| Marker | **CRC_**  **median** | **Control_**  **median** | **Fold Change** | **p_value** | **p_value^adj^** | **AUC^*^**  **(95% CI)** | **AUC^BS^**  **(95% CI)** | **Se^BS^ % at**  **80% Sp** | **Se^BS^ % at**  **90% Sp** | **CRC_**  **median** | **Control_**  **median** | **Fold Change** | **p_value** | **p_value^adj^** | **AUC^*^**  **(95% CI)** | **AUC^BS^**  **(95% CI)** | **Se^BS^ % at**  **80% Sp** | **Se^BS^ % at**  **90% Sp** |
| MGMT | 4.53 | 4.80 | -0.56 | 0.19 | 0.35 | 0.55[0.47-0.64] | 0.5[0.35-0.64] | 19% | 9% | 5.00 | 4.21 | 1.57 | 0.15 | 0.34 | 0.57[0.48-0.66] | 0.52[0.33-0.67] | 14% | 6% |
| MIA | 10.05 | 10.02 | 0.07 | 0.49 | 0.64 | 0.53[0.45-0.61] | 0.48[0.38-0.6] | 19% | 12% | 10.00 | 10.06 | -0.13 | 0.10 | 0.26 | 0.58[0.48-0.67] | 0.51[0.33-0.67] | 21% | 11% |
| MIC.A.B | 4.02 | 3.65 | 0.75 | 0.01 | 0.05 | 0.6[0.52-0.68] | 0.51[0.32-0.68] | 25% | 12% | 3.95 | 4.00 | -0.10 | 0.51 | 0.73 | 0.47[0.38-0.56] | 0.47[0.36-0.61] | 14% | 7% |
| MILR1 | 1.97 | 1.93 | 0.09 | 0.33 | 0.49 | 0.54[0.46-0.62] | 0.5[0.37-0.62] | 23% | 11% | 1.89 | 2.01 | -0.23 | 0.09 | 0.25 | 0.58[0.49-0.67] | 0.53[0.34-0.69] | 26% | 15% |
| MK | 6.98 | 6.75 | 0.46 | 0.00 | 0.01 | 0.63[0.55-0.71] | 0.61[0.52-0.73] | 38% | 23% | 6.90 | 6.68 | 0.45 | 0.03 | 0.16 | 0.6[0.51-0.69] | 0.56[0.46-0.7] | 24% | 13% |
| MMP.2 | 3.59 | 3.66 | -0.15 | 0.05 | 0.13 | 0.58[0.5-0.66] | 0.54[0.46-0.68] | 26% | 15% | 3.69 | 3.66 | 0.06 | 0.40 | 0.63 | 0.54[0.45-0.63] | 0.49[0.35-0.63] | 19% | 9% |
| MMP.3 | 6.28 | 6.20 | 0.15 | 0.31 | 0.46 | 0.54[0.46-0.62] | 0.5[0.37-0.63] | 20% | 12% | 6.23 | 6.28 | -0.11 | 0.60 | 0.75 | 0.53[0.43-0.62] | 0.46[0.36-0.58] | 16% | 7% |
| MMP.9 | 5.54 | 5.53 | 0.02 | 0.30 | 0.46 | 0.54[0.46-0.62] | 0.5[0.38-0.63] | 18% | 11% | 5.63 | 5.53 | 0.21 | 0.05 | 0.17 | 0.59[0.5-0.69] | 0.55[0.46-0.71] | 29% | 16% |
| MPO | 3.87 | 3.64 | 0.45 | 0.00 | 0.01 | 0.63[0.55-0.71] | 0.6[0.52-0.73] | 30% | 17% | 3.85 | 3.73 | 0.24 | 0.53 | 0.73 | 0.53[0.44-0.62] | 0.46[0.35-0.57] | 15% | 6% |
| MSLN | 2.66 | 2.76 | -0.19 | 0.65 | 0.76 | 0.52[0.44-0.6] | 0.45[0.36-0.56] | 16% | 9% | 2.72 | 2.64 | 0.16 | 0.37 | 0.63 | 0.54[0.45-0.64] | 0.5[0.35-0.65] | 24% | 13% |
| MUC.16 | 3.33 | 3.33 | -0.01 | 0.44 | 0.60 | 0.47[0.39-0.55] | 0.5[0.38-0.62] | 21% | 10% | 3.38 | 3.30 | 0.16 | 0.22 | 0.44 | 0.56[0.47-0.65] | 0.5[0.34-0.66] | 22% | 12% |
| NCR1 | 1.56 | 1.54 | 0.05 | 0.77 | 0.85 | 0.51[0.43-0.59] | 0.47[0.38-0.57] | 19% | 7% | 1.55 | 1.53 | 0.03 | 0.84 | 0.90 | 0.51[0.41-0.6] | 0.45[0.35-0.58] | 16% | 6% |
| NF2 | -0.17 | 0.12 | -0.59 | 0.26 | 0.42 | 0.55[0.46-0.63] | 0.5[0.37-0.64] | 22% | 9% | 0.08 | -0.08 | 0.32 | 0.03 | 0.16 | 0.59[0.51-0.67] | 0.46[0.3-0.63] | 14% | 7% |
| NFATC3 | 0.05 | 0.15 | -0.21 | 0.12 | 0.25 | 0.56[0.48-0.65] | 0.53[0.43-0.66] | 25% | 10% | 0.08 | 0.07 | 0.01 | 0.64 | 0.79 | 0.52[0.43-0.61] | 0.45[0.35-0.58] | 15% | 5% |
| Notch.3 | 3.64 | 3.79 | -0.29 | 0.03 | 0.08 | 0.59[0.51-0.67] | 0.55[0.47-0.68] | 23% | 10% | 3.74 | 3.73 | 0.03 | 0.34 | 0.59 | 0.45[0.36-0.55] | 0.49[0.34-0.63] | 17% | 8% |
| NT.proBNP | 3.42 | 2.87 | 1.11 | 0.00 | 0.01 | 0.64[0.56-0.71] | 0.62[0.54-0.75] | 37% | 23% | 3.11 | 3.02 | 0.18 | 0.30 | 0.55 | 0.55[0.45-0.64] | 0.5[0.34-0.64] | 24% | 12% |
| NTF4 | 0.49 | 0.55 | -0.12 | 0.95 | 0.96 | 0.5[0.42-0.58] | 0.47[0.38-0.57] | 16% | 10% | 0.50 | 0.50 | 0.00 | 0.57 | 0.74 | 0.47[0.38-0.57] | 0.47[0.37-0.61] | 13% | 7% |
| OPG | 3.67 | 3.54 | 0.26 | 0.01 | 0.04 | 0.61[0.53-0.69] | 0.58[0.49-0.71] | 32% | 20% | 3.74 | 3.59 | 0.30 | 0.04 | 0.17 | 0.6[0.5-0.69] | 0.56[0.45-0.72] | 32% | 21% |
| OPN | 6.23 | 5.80 | 0.84 | 0.00 | 0.00 | 0.75[0.68-0.81] | 0.73[0.66-0.84] | 52% | 37% | 6.29 | 6.01 | 0.56 | 0.01 | 0.11 | 0.62[0.53-0.71] | 0.59[0.49-0.73] | 32% | 18% |
| PADI2 | 0.13 | 0.18 | -0.10 | 0.74 | 0.84 | 0.51[0.43-0.59] | 0.46[0.38-0.56] | 20% | 10% | 0.15 | 0.14 | 0.02 | 0.36 | 0.62 | 0.54[0.46-0.62] | 0.46[0.35-0.57] | 15% | 7% |
| PAI | 6.73 | 6.65 | 0.16 | 0.12 | 0.26 | 0.56[0.48-0.65] | 0.52[0.37-0.65] | 23% | 13% | 6.71 | 6.56 | 0.30 | 0.33 | 0.58 | 0.55[0.46-0.64] | 0.49[0.36-0.65] | 18% | 6% |
| PCSK9 | 2.20 | 2.18 | 0.04 | 0.87 | 0.92 | 0.51[0.42-0.59] | 0.47[0.38-0.57] | 18% | 10% | 2.24 | 2.19 | 0.10 | 0.54 | 0.73 | 0.53[0.44-0.62] | 0.47[0.35-0.59] | 15% | 7% |
| PDGF.subunit.A | 5.72 | 5.69 | 0.06 | 0.52 | 0.66 | 0.53[0.44-0.61] | 0.47[0.37-0.58] | 20% | 7% | 5.68 | 5.53 | 0.29 | 0.98 | 0.98 | 0.5[0.41-0.59] | 0.45[0.36-0.56] | 15% | 5% |
| PECAM.1 | 4.30 | 4.37 | -0.15 | 0.19 | 0.35 | 0.55[0.47-0.64] | 0.5[0.37-0.63] | 19% | 11% | 4.36 | 4.31 | 0.11 | 0.54 | 0.73 | 0.53[0.44-0.62] | 0.46[0.35-0.6] | 17% | 6% |
| PGLYRP1 | 8.14 | 8.00 | 0.29 | 0.16 | 0.32 | 0.56[0.48-0.64] | 0.51[0.37-0.65] | 19% | 10% | 8.16 | 7.97 | 0.37 | 0.21 | 0.42 | 0.56[0.47-0.65] | 0.49[0.33-0.65] | 15% | 9% |
| PI3 | 3.39 | 3.24 | 0.31 | 0.02 | 0.06 | 0.6[0.52-0.68] | 0.56[0.49-0.69] | 30% | 19% | 3.46 | 3.36 | 0.19 | 0.83 | 0.89 | 0.51[0.41-0.61] | 0.47[0.36-0.6] | 22% | 9% |
| PIK3AP1 | 3.70 | 4.06 | -0.72 | 0.34 | 0.50 | 0.54[0.46-0.62] | 0.49[0.36-0.62] | 16% | 8% | 4.13 | 3.64 | 0.98 | 0.06 | 0.19 | 0.59[0.5-0.68] | 0.53[0.35-0.69] | 21% | 8% |
| PLC | 6.42 | 6.46 | -0.06 | 0.72 | 0.82 | 0.51[0.43-0.6] | 0.46[0.37-0.56] | 17% | 9% | 6.42 | 6.49 | -0.14 | 0.30 | 0.54 | 0.55[0.45-0.65] | 0.48[0.34-0.62] | 17% | 9% |
|  | **DISCOVERY SET** | | | | | | | | | **VALIDATION SET** | | | | | | | | |
| Marker | **CRC_**  **median** | **Control_**  **median** | **Fold Change** | **p_value** | **p_value^adj^** | **AUC^*^**  **(95% CI)** | **AUC^BS^**  **(95% CI)** | **Se^BS^ % at**  **80% Sp** | **Se^BS^ % at**  **90% Sp** | **CRC_**  **median** | **Control_**  **median** | **Fold Change** | **p_value** | **p_value^adj^** | **AUC^*^**  **(95% CI)** | **AUC^BS^**  **(95% CI)** | **Se^BS^ % at**  **80% Sp** | **Se^BS^ % at**  **90% Sp** |
| PLXNA4 | 5.30 | 5.47 | -0.34 | 0.13 | 0.27 | 0.56[0.48-0.64] | 0.51[0.35-0.64] | 22% | 13% | 5.70 | 5.09 | 1.21 | 0.06 | 0.19 | 0.59[0.5-0.68] | 0.53[0.32-0.69] | 22% | 7% |
| PODXL | 3.33 | 3.43 | -0.21 | 0.00 | 0.00 | 0.65[0.57-0.72] | 0.63[0.55-0.74] | 34% | 23% | 3.34 | 3.46 | -0.24 | 0.01 | 0.10 | 0.63[0.54-0.71] | 0.6[0.51-0.74] | 27% | 13% |
| PON3 | 4.95 | 5.58 | -1.26 | 0.00 | 0.00 | 0.75[0.68-0.82] | 0.74[0.65-0.84] | 55% | 41% | 5.28 | 5.44 | -0.33 | 0.05 | 0.17 | 0.59[0.51-0.68] | 0.54[0.37-0.69] | 23% | 11% |
| PPP1R9B | 4.40 | 4.61 | -0.42 | 0.89 | 0.92 | 0.51[0.42-0.59] | 0.46[0.38-0.55] | 16% | 6% | 4.66 | 4.09 | 1.15 | 0.08 | 0.24 | 0.58[0.49-0.67] | 0.52[0.34-0.68] | 18% | 7% |
| PPY | 7.89 | 7.30 | 1.17 | 0.06 | 0.14 | 0.58[0.5-0.66] | 0.54[0.44-0.68] | 23% | 11% | 7.70 | 7.60 | 0.21 | 0.92 | 0.95 | 0.5[0.41-0.6] | 0.46[0.36-0.56] | 15% | 6% |
| PRDX1 | 2.41 | 2.70 | -0.59 | 0.02 | 0.06 | 0.6[0.52-0.68] | 0.57[0.49-0.7] | 23% | 12% | 2.73 | 2.50 | 0.45 | 0.29 | 0.54 | 0.55[0.46-0.64] | 0.48[0.33-0.63] | 17% | 7% |
| PRDX3 | 0.08 | 0.08 | 0.00 | 0.37 | 0.52 | 0.54[0.46-0.62] | 0.48[0.36-0.61] | 20% | 8% | 0.21 | -0.02 | 0.48 | 0.01 | 0.10 | 0.63[0.54-0.72] | 0.58[0.5-0.74] | 34% | 18% |
| PRDX5 | 6.57 | 6.51 | 0.13 | 0.25 | 0.41 | 0.55[0.47-0.63] | 0.51[0.37-0.63] | 18% | 9% | 6.77 | 6.44 | 0.66 | 0.10 | 0.26 | 0.58[0.49-0.67] | 0.52[0.34-0.68] | 24% | 8% |
| PRKCQ | 0.23 | 0.23 | -0.01 | 0.57 | 0.70 | 0.52[0.45-0.6] | 0.48[0.37-0.59] | 19% | 7% | 0.23 | 0.23 | 0.00 | 0.35 | 0.61 | 0.47[0.4-0.53] | 0.47[0.39-0.6] | 18% | 9% |
| PRTN3 | 5.05 | 4.63 | 0.83 | 0.00 | 0.00 | 0.65[0.57-0.72] | 0.62[0.54-0.75] | 33% | 19% | 5.07 | 4.74 | 0.66 | 0.06 | 0.19 | 0.59[0.5-0.68] | 0.49[0.31-0.65] | 15% | 5% |
| PSIP1 | 2.69 | 2.58 | 0.22 | 0.95 | 0.96 | 0.5[0.42-0.58] | 0.46[0.37-0.55] | 16% | 6% | 3.04 | 2.31 | 1.47 | 0.00 | 0.06 | 0.65[0.56-0.73] | 0.58[0.5-0.75] | 28% | 12% |
| PSP.D | 1.93 | 2.22 | -0.59 | 0.02 | 0.07 | 0.59[0.51-0.67] | 0.55[0.41-0.69] | 28% | 15% | 2.16 | 2.03 | 0.27 | 0.56 | 0.74 | 0.53[0.43-0.62] | 0.46[0.34-0.59] | 16% | 9% |
| PTH1R | 1.48 | 1.42 | 0.12 | 0.30 | 0.46 | 0.54[0.46-0.62] | 0.47[0.34-0.59] | 15% | 8% | 1.42 | 1.46 | -0.08 | 0.41 | 0.64 | 0.54[0.45-0.63] | 0.46[0.35-0.6] | 16% | 9% |
| PVRL4 | 4.94 | 4.83 | 0.22 | 0.00 | 0.02 | 0.62[0.54-0.7] | 0.59[0.51-0.72] | 35% | 15% | 4.87 | 4.91 | -0.08 | 0.68 | 0.82 | 0.52[0.42-0.62] | 0.46[0.35-0.57] | 15% | 7% |
| RARRES2 | 11.59 | 11.42 | 0.33 | 0.00 | 0.00 | 0.72[0.65-0.79] | 0.56[0.28-0.76] | 28% | 17% | 11.58 | 11.45 | 0.26 | 0.00 | 0.07 | 0.64[0.55-0.73] | 0.6[0.52-0.74] | 32% | 13% |
| RET | 3.90 | 4.08 | -0.37 | 0.00 | 0.02 | 0.62[0.54-0.7] | 0.6[0.52-0.72] | 34% | 23% | 3.82 | 4.05 | -0.47 | 0.02 | 0.16 | 0.61[0.52-0.69] | 0.58[0.49-0.72] | 24% | 11% |
| RETN | 7.02 | 6.72 | 0.60 | 0.04 | 0.10 | 0.59[0.51-0.67] | 0.55[0.47-0.68] | 26% | 12% | 6.93 | 6.76 | 0.34 | 0.03 | 0.16 | 0.6[0.51-0.69] | 0.54[0.4-0.71] | 23% | 12% |
| RSPO3 | 3.67 | 3.59 | 0.16 | 0.92 | 0.94 | 0.5[0.42-0.59] | 0.46[0.38-0.55] | 15% | 8% | 3.61 | 3.72 | -0.21 | 0.53 | 0.73 | 0.53[0.44-0.62] | 0.46[0.34-0.58] | 15% | 6% |
| S100A11 | 4.15 | 4.06 | 0.18 | 0.30 | 0.46 | 0.54[0.46-0.62] | 0.5[0.37-0.62] | 20% | 10% | 4.31 | 3.94 | 0.74 | 0.03 | 0.16 | 0.6[0.51-0.69] | 0.55[0.39-0.71] | 24% | 10% |
| S100A4 | 2.07 | 2.35 | -0.56 | 0.00 | 0.00 | 0.65[0.58-0.73] | 0.64[0.55-0.76] | 34% | 12% | 2.13 | 2.25 | -0.24 | 0.16 | 0.37 | 0.57[0.48-0.66] | 0.51[0.36-0.66] | 17% | 9% |
| SCAMP3 | 5.12 | 5.14 | -0.04 | 0.46 | 0.61 | 0.47[0.39-0.55] | 0.48[0.36-0.6] | 18% | 9% | 5.21 | 4.90 | 0.62 | 0.39 | 0.63 | 0.54[0.45-0.63] | 0.47[0.35-0.62] | 17% | 8% |
| SCF | 8.82 | 8.96 | -0.29 | 0.02 | 0.07 | 0.59[0.51-0.67] | 0.56[0.48-0.69] | 29% | 17% | 8.81 | 8.97 | -0.33 | 0.13 | 0.33 | 0.57[0.48-0.66] | 0.5[0.32-0.65] | 22% | 8% |
| SCGB3A2 | 2.22 | 2.17 | 0.11 | 0.49 | 0.64 | 0.53[0.45-0.61] | 0.49[0.37-0.61] | 22% | 11% | 2.26 | 2.15 | 0.22 | 0.38 | 0.63 | 0.54[0.45-0.63] | 0.48[0.34-0.62] | 18% | 9% |
| SELE | 12.27 | 12.20 | 0.12 | 0.50 | 0.65 | 0.53[0.45-0.61] | 0.48[0.37-0.59] | 17% | 8% | 12.54 | 12.20 | 0.68 | 0.04 | 0.17 | 0.6[0.51-0.69] | 0.53[0.33-0.7] | 24% | 12% |
| SELP | 10.36 | 10.47 | -0.22 | 0.59 | 0.72 | 0.52[0.44-0.6] | 0.47[0.37-0.58] | 17% | 7% | 10.32 | 10.26 | 0.12 | 0.83 | 0.89 | 0.49[0.4-0.58] | 0.47[0.37-0.59] | 15% | 5% |
| SEZ6L | 3.25 | 3.42 | -0.33 | 0.00 | 0.01 | 0.63[0.55-0.71] | 0.61[0.53-0.73] | 35% | 22% | 3.33 | 3.48 | -0.30 | 0.00 | 0.04 | 0.65[0.56-0.74] | 0.63[0.54-0.77] | 40% | 22% |
| SH2B3 | 4.44 | 4.52 | -0.16 | 0.29 | 0.46 | 0.54[0.46-0.63] | 0.49[0.36-0.62] | 21% | 11% | 4.69 | 4.06 | 1.25 | 0.14 | 0.34 | 0.57[0.48-0.66] | 0.5[0.33-0.65] | 16% | 6% |
| SH2D1A | 1.94 | 1.97 | -0.06 | 0.12 | 0.25 | 0.56[0.48-0.65] | 0.53[0.42-0.66] | 18% | 9% | 2.40 | 1.61 | 1.59 | 0.00 | 0.08 | 0.64[0.55-0.72] | 0.6[0.51-0.75] | 36% | 13% |
|  | **DISCOVERY SET** | | | | | | | | | **VALIDATION SET** | | | | | | | | |
| Marker | **CRC_**  **median** | **Control_**  **median** | **Fold Change** | **p_value** | **p_value^adj^** | **AUC^*^**  **(95% CI)** | **AUC^BS^**  **(95% CI)** | **Se^BS^ % at**  **80% Sp** | **Se^BS^ % at**  **90% Sp** | **CRC_**  **median** | **Control_**  **median** | **Fold Change** | **p_value** | **p_value^adj^** | **AUC^*^**  **(95% CI)** | **AUC^BS^**  **(95% CI)** | **Se^BS^ % at**  **80% Sp** | **Se^BS^ % at**  **90% Sp** |
| SHPS.1 | 3.08 | 3.10 | -0.03 | 0.48 | 0.63 | 0.47[0.39-0.55] | 0.48[0.36-0.59] | 17% | 9% | 3.20 | 3.07 | 0.25 | 0.07 | 0.21 | 0.59[0.49-0.68] | 0.54[0.42-0.7] | 23% | 12% |
| SIT1 | 3.37 | 3.04 | 0.66 | 0.03 | 0.08 | 0.59[0.51-0.67] | 0.56[0.47-0.69] | 26% | 14% | 3.47 | 2.76 | 1.41 | 0.00 | 0.04 | 0.65[0.57-0.74] | 0.63[0.54-0.77] | 34% | 17% |
| SPARC | 6.11 | 6.08 | 0.05 | 0.33 | 0.48 | 0.54[0.46-0.62] | 0.49[0.36-0.61] | 18% | 10% | 6.07 | 6.10 | -0.06 | 0.41 | 0.64 | 0.54[0.45-0.63] | 0.48[0.36-0.61] | 15% | 5% |
| SPON1 | 0.92 | 0.89 | 0.05 | 0.40 | 0.56 | 0.54[0.45-0.62] | 0.49[0.37-0.61] | 19% | 11% | 0.90 | 0.90 | 0.00 | 0.82 | 0.89 | 0.49[0.41-0.57] | 0.47[0.38-0.58] | 14% | 7% |
| SPRY2 | 1.83 | 1.81 | 0.05 | 0.64 | 0.75 | 0.48[0.4-0.56] | 0.47[0.37-0.58] | 19% | 7% | 1.96 | 1.62 | 0.68 | 0.00 | 0.08 | 0.63[0.54-0.71] | 0.54[0.33-0.71] | 26% | 10% |
| SRPK2 | 0.74 | 1.02 | -0.57 | 0.15 | 0.30 | 0.56[0.48-0.64] | 0.51[0.36-0.64] | 20% | 8% | 0.93 | 0.63 | 0.60 | 0.01 | 0.10 | 0.63[0.54-0.71] | 0.52[0.28-0.71] | 20% | 7% |
| ST2 | 3.53 | 3.43 | 0.19 | 0.03 | 0.07 | 0.59[0.51-0.67] | 0.56[0.48-0.69] | 32% | 20% | 3.61 | 3.51 | 0.21 | 0.16 | 0.36 | 0.57[0.47-0.66] | 0.5[0.33-0.66] | 21% | 11% |
| STC1 | 5.42 | 5.34 | 0.17 | 0.19 | 0.35 | 0.55[0.47-0.64] | 0.5[0.37-0.63] | 21% | 13% | 5.54 | 5.40 | 0.27 | 0.20 | 0.42 | 0.56[0.47-0.65] | 0.51[0.35-0.66] | 20% | 13% |
| SYND1 | 6.37 | 6.18 | 0.38 | 0.07 | 0.16 | 0.58[0.49-0.66] | 0.56[0.47-0.68] | 36% | 24% | 6.19 | 6.29 | -0.20 | 0.40 | 0.63 | 0.54[0.44-0.64] | 0.47[0.33-0.61] | 16% | 8% |
| t.PA | 6.78 | 6.55 | 0.45 | 0.01 | 0.04 | 0.6[0.52-0.68] | 0.58[0.49-0.7] | 35% | 19% | 6.79 | 6.62 | 0.36 | 0.04 | 0.17 | 0.6[0.5-0.69] | 0.55[0.37-0.7] | 29% | 17% |
| TANK | 1.65 | 1.74 | -0.19 | 0.30 | 0.46 | 0.54[0.46-0.62] | 0.49[0.35-0.62] | 19% | 6% | 1.76 | 1.44 | 0.64 | 0.03 | 0.16 | 0.6[0.51-0.69] | 0.54[0.35-0.7] | 26% | 12% |
| TCL1A | 5.76 | 6.55 | -1.58 | 0.00 | 0.00 | 0.65[0.57-0.73] | 0.64[0.55-0.75] | 32% | 17% | 6.32 | 5.92 | 0.80 | 0.25 | 0.48 | 0.55[0.46-0.65] | 0.5[0.33-0.64] | 21% | 10% |
| TFF3 | 5.13 | 4.77 | 0.73 | 0.00 | 0.00 | 0.75[0.68-0.82] | 0.74[0.66-0.84] | 49% | 36% | 5.10 | 4.89 | 0.42 | 0.01 | 0.09 | 0.63[0.54-0.72] | 0.58[0.51-0.75] | 31% | 18% |
| TFPI | 9.05 | 9.07 | -0.03 | 0.91 | 0.94 | 0.5[0.41-0.58] | 0.46[0.38-0.55] | 17% | 9% | 9.18 | 9.03 | 0.31 | 0.00 | 0.02 | 0.67[0.58-0.75] | 0.64[0.55-0.78] | 34% | 19% |
| TFPI.2 | 7.93 | 7.66 | 0.55 | 0.00 | 0.00 | 0.68[0.61-0.76] | 0.67[0.57-0.78] | 43% | 27% | 7.82 | 7.71 | 0.21 | 0.05 | 0.17 | 0.59[0.5-0.68] | 0.55[0.43-0.71] | 27% | 15% |
| TGF.alpha | 3.44 | 3.24 | 0.39 | 0.08 | 0.18 | 0.57[0.49-0.65] | 0.52[0.41-0.66] | 21% | 12% | 3.67 | 3.17 | 0.99 | 0.01 | 0.11 | 0.62[0.53-0.71] | 0.58[0.49-0.73] | 30% | 15% |
| TGFR.2 | 6.53 | 6.39 | 0.28 | 0.00 | 0.01 | 0.63[0.55-0.7] | 0.61[0.53-0.73] | 32% | 19% | 6.36 | 6.50 | -0.27 | 0.07 | 0.21 | 0.59[0.49-0.68] | 0.53[0.34-0.69] | 24% | 14% |
| TIMP4 | 3.43 | 3.36 | 0.15 | 0.25 | 0.41 | 0.55[0.47-0.63] | 0.51[0.36-0.63] | 23% | 12% | 3.46 | 3.39 | 0.13 | 0.47 | 0.70 | 0.53[0.44-0.63] | 0.48[0.34-0.63] | 24% | 10% |
| TLR3 | 5.11 | 5.15 | -0.09 | 0.30 | 0.46 | 0.54[0.46-0.62] | 0.51[0.38-0.64] | 24% | 14% | 5.08 | 5.09 | -0.02 | 0.18 | 0.39 | 0.44[0.34-0.53] | 0.51[0.39-0.67] | 20% | 9% |
| TLT.2 | 4.65 | 4.66 | -0.01 | 0.79 | 0.86 | 0.49[0.41-0.57] | 0.47[0.38-0.59] | 22% | 14% | 4.62 | 4.67 | -0.09 | 0.42 | 0.65 | 0.54[0.45-0.63] | 0.49[0.34-0.64] | 18% | 7% |
| TNF.R1 | 5.77 | 5.52 | 0.48 | 0.00 | 0.00 | 0.68[0.6-0.76] | 0.66[0.58-0.77] | 42% | 30% | 5.68 | 5.62 | 0.13 | 0.25 | 0.48 | 0.55[0.46-0.65] | 0.49[0.36-0.66] | 19% | 11% |
| TNF.R2 | 4.89 | 4.64 | 0.49 | 0.00 | 0.00 | 0.68[0.61-0.76] | 0.67[0.58-0.78] | 48% | 28% | 4.86 | 4.78 | 0.17 | 0.20 | 0.42 | 0.56[0.47-0.65] | 0.49[0.34-0.65] | 17% | 9% |
| TNFRSF10C | 5.58 | 5.55 | 0.06 | 0.56 | 0.69 | 0.52[0.44-0.61] | 0.48[0.37-0.59] | 17% | 9% | 5.49 | 5.55 | -0.10 | 0.53 | 0.73 | 0.53[0.44-0.62] | 0.46[0.34-0.58] | 16% | 6% |
| TNFRSF14 | 4.65 | 4.60 | 0.09 | 0.11 | 0.24 | 0.57[0.49-0.65] | 0.53[0.44-0.67] | 30% | 17% | 4.61 | 4.65 | -0.09 | 0.37 | 0.63 | 0.54[0.45-0.63] | 0.49[0.35-0.64] | 17% | 8% |
| TNFRSF19 | 3.79 | 3.77 | 0.05 | 0.41 | 0.57 | 0.53[0.45-0.62] | 0.49[0.38-0.62] | 23% | 12% | 3.72 | 3.87 | -0.31 | 0.05 | 0.18 | 0.59[0.5-0.69] | 0.51[0.31-0.68] | 22% | 12% |
| TNFRSF4 | 3.26 | 3.00 | 0.52 | 0.02 | 0.06 | 0.6[0.52-0.68] | 0.57[0.49-0.7] | 32% | 18% | 3.04 | 3.08 | -0.08 | 0.48 | 0.71 | 0.53[0.44-0.63] | 0.47[0.33-0.6] | 16% | 7% |
| TNFRSF6B | 4.73 | 4.42 | 0.62 | 0.00 | 0.01 | 0.63[0.55-0.71] | 0.61[0.53-0.73] | 37% | 20% | 4.62 | 4.52 | 0.20 | 0.48 | 0.71 | 0.53[0.44-0.63] | 0.49[0.36-0.63] | 20% | 11% |
| TNFSF13 | 8.53 | 8.32 | 0.41 | 0.00 | 0.02 | 0.62[0.54-0.7] | 0.59[0.52-0.72] | 30% | 15% | 8.46 | 8.39 | 0.15 | 0.23 | 0.46 | 0.56[0.46-0.65] | 0.49[0.33-0.64] | 18% | 9% |
|  | **DISCOVERY SET** | | | | | | | | | **VALIDATION SET** | | | | | | | | |
| Marker | **CRC_**  **median** | **Control_**  **median** | **Fold Change** | **p_value** | **p_value^adj^** | **AUC^*^**  **(95% CI)** | **AUC^BS^**  **(95% CI)** | **Se^BS^ % at**  **80% Sp** | **Se^BS^ % at**  **90% Sp** | **CRC_**  **median** | **Control_**  **median** | **Fold Change** | **p_value** | **p_value^adj^** | **AUC^*^**  **(95% CI)** | **AUC^BS^**  **(95% CI)** | **Se^BS^ % at**  **80% Sp** | **Se^BS^ % at**  **90% Sp** |
| TNFSF13B | 6.61 | 6.50 | 0.21 | 0.04 | 0.12 | 0.58[0.5-0.66] | 0.53[0.41-0.67] | 22% | 11% | 6.63 | 6.51 | 0.24 | 0.06 | 0.19 | 0.59[0.5-0.68] | 0.54[0.46-0.71] | 24% | 13% |
| TPSAB1 | 3.04 | 3.06 | -0.05 | 0.51 | 0.65 | 0.53[0.45-0.61] | 0.48[0.36-0.6] | 16% | 7% | 2.90 | 3.17 | -0.55 | 0.06 | 0.19 | 0.59[0.49-0.69] | 0.51[0.3-0.67] | 23% | 13% |
| TR | 5.32 | 4.87 | 0.89 | 0.00 | 0.00 | 0.69[0.62-0.77] | 0.68[0.6-0.79] | 46% | 35% | 5.27 | 4.84 | 0.86 | 0.00 | 0.00 | 0.74[0.66-0.82] | 0.72[0.64-0.85] | 50% | 33% |
| TR.AP | 3.57 | 3.71 | -0.28 | 0.01 | 0.05 | 0.6[0.52-0.68] | 0.57[0.49-0.7] | 32% | 22% | 3.63 | 3.67 | -0.08 | 0.69 | 0.83 | 0.52[0.43-0.61] | 0.45[0.35-0.57] | 15% | 6% |
| TRAF2 | 2.11 | 2.26 | -0.30 | 0.34 | 0.50 | 0.54[0.46-0.62] | 0.47[0.36-0.59] | 14% | 8% | 2.13 | 2.06 | 0.15 | 0.46 | 0.70 | 0.47[0.37-0.56] | 0.48[0.35-0.63] | 22% | 10% |
| TRAIL | 7.45 | 7.63 | -0.36 | 0.00 | 0.00 | 0.66[0.58-0.73] | 0.64[0.55-0.75] | 37% | 25% | 7.46 | 7.65 | -0.38 | 0.00 | 0.03 | 0.66[0.57-0.75] | 0.64[0.55-0.78] | 32% | 17% |
| TREM1 | 1.62 | 1.58 | 0.07 | 0.19 | 0.35 | 0.55[0.47-0.63] | 0.51[0.38-0.64] | 25% | 12% | 1.66 | 1.64 | 0.05 | 0.47 | 0.70 | 0.53[0.44-0.63] | 0.48[0.35-0.62] | 20% | 8% |
| TRIM21 | 1.83 | 2.00 | -0.35 | 0.45 | 0.61 | 0.53[0.45-0.61] | 0.48[0.36-0.61] | 20% | 7% | 2.02 | 1.71 | 0.63 | 0.01 | 0.10 | 0.62[0.53-0.71] | 0.52[0.31-0.71] | 20% | 9% |
| TRIM5 | 1.33 | 1.37 | -0.08 | 0.89 | 0.92 | 0.51[0.42-0.59] | 0.46[0.37-0.55] | 16% | 8% | 1.28 | 1.23 | 0.10 | 0.57 | 0.74 | 0.53[0.44-0.62] | 0.45[0.35-0.56] | 15% | 6% |
| TXLNA | 5.92 | 5.85 | 0.14 | 0.88 | 0.92 | 0.51[0.42-0.59] | 0.46[0.38-0.55] | 16% | 7% | 6.01 | 5.50 | 1.02 | 0.03 | 0.16 | 0.6[0.52-0.69] | 0.55[0.46-0.71] | 21% | 8% |
| U.PAR | 5.10 | 4.83 | 0.54 | 0.01 | 0.03 | 0.61[0.53-0.69] | 0.58[0.49-0.71] | 27% | 11% | 5.00 | 4.89 | 0.21 | 0.05 | 0.19 | 0.59[0.5-0.68] | 0.54[0.37-0.7] | 24% | 11% |
| uPA | 4.89 | 4.94 | -0.09 | 0.21 | 0.36 | 0.55[0.47-0.63] | 0.5[0.35-0.63] | 21% | 13% | 4.93 | 4.92 | 0.01 | 0.54 | 0.73 | 0.47[0.38-0.56] | 0.47[0.35-0.61] | 17% | 7% |
| VEGFA | 10.16 | 9.69 | 0.93 | 0.00 | 0.00 | 0.66[0.59-0.74] | 0.64[0.56-0.76] | 37% | 23% | 9.99 | 9.75 | 0.48 | 0.09 | 0.24 | 0.58[0.49-0.67] | 0.51[0.33-0.67] | 17% | 8% |
| VEGFR.2 | 6.93 | 7.06 | -0.25 | 0.01 | 0.04 | 0.61[0.53-0.69] | 0.58[0.5-0.7] | 33% | 15% | 7.03 | 7.01 | 0.05 | 0.70 | 0.84 | 0.48[0.39-0.58] | 0.47[0.35-0.6] | 19% | 9% |
| VEGFR.3 | 6.94 | 6.88 | 0.13 | 0.05 | 0.13 | 0.58[0.5-0.66] | 0.54[0.44-0.67] | 25% | 13% | 6.88 | 6.96 | -0.16 | 0.05 | 0.18 | 0.59[0.5-0.68] | 0.54[0.36-0.69] | 23% | 10% |
| VIM | 5.34 | 5.15 | 0.37 | 0.18 | 0.34 | 0.56[0.47-0.64] | 0.52[0.36-0.65] | 21% | 10% | 5.51 | 5.19 | 0.64 | 0.02 | 0.12 | 0.61[0.52-0.7] | 0.58[0.49-0.73] | 30% | 11% |
| vWF | 7.03 | 6.75 | 0.56 | 0.03 | 0.09 | 0.59[0.51-0.67] | 0.55[0.46-0.68] | 29% | 16% | 6.72 | 6.82 | -0.19 | 0.17 | 0.38 | 0.56[0.47-0.66] | 0.5[0.34-0.66] | 23% | 14% |
| WFDC2 | 7.77 | 7.55 | 0.43 | 0.00 | 0.00 | 0.67[0.6-0.75] | 0.66[0.57-0.77] | 40% | 29% | 7.73 | 7.76 | -0.06 | 0.98 | 0.98 | 0.5[0.41-0.59] | 0.46[0.36-0.56] | 16% | 7% |
| WIF.1 | 5.39 | 5.29 | 0.19 | 0.19 | 0.35 | 0.55[0.47-0.64] | 0.5[0.36-0.63] | 21% | 12% | 5.24 | 5.31 | -0.15 | 0.27 | 0.51 | 0.55[0.46-0.64] | 0.48[0.34-0.63] | 18% | 6% |
| WISP.1 | 5.45 | 5.33 | 0.25 | 0.02 | 0.06 | 0.6[0.52-0.68] | 0.57[0.48-0.69] | 32% | 16% | 5.23 | 5.40 | -0.34 | 0.05 | 0.18 | 0.59[0.5-0.68] | 0.55[0.45-0.7] | 25% | 12% |
| X5..NT | 9.94 | 9.80 | 0.28 | 0.13 | 0.27 | 0.56[0.48-0.64] | 0.52[0.41-0.65] | 22% | 12% | 9.83 | 9.90 | -0.12 | 0.79 | 0.88 | 0.51[0.42-0.61] | 0.46[0.35-0.57] | 16% | 7% |
| XPNPEP2 | 7.35 | 7.60 | -0.49 | 0.00 | 0.02 | 0.62[0.54-0.7] | 0.55[0.5-0.71] | 24% | 11% | 7.51 | 7.48 | 0.07 | 0.97 | 0.98 | 0.5[0.41-0.59] | 0.45[0.36-0.56] | 15% | 6% |
| ZBTB16 | 1.38 | 1.54 | -0.32 | 0.26 | 0.42 | 0.55[0.47-0.63] | 0.48[0.36-0.61] | 17% | 8% | 1.49 | 1.40 | 0.17 | 0.49 | 0.71 | 0.53[0.44-0.62] | 0.45[0.34-0.57] | 15% | 6% |
| CDKN1A | - | - | - | - | - | - | - | - | - | 3.72 | 3.08 | 1.27 | 0.03 | 0.16 | 0.6[0.51-0.69] | 0.54[0.35-0.69] | 21% | 6% |
| DKN1A | 4.42 | 4.32 | 0.20 | 0.28 | 0.45 | 0.54[0.46-0.63] | 0.5[0.36-0.63] | 23% | 15% | - | - | - | - | - | - | - | - | - |
| GP6 | - | - | - | - | - | - | - | - | - | 2.5 | 2.4 | 0.13 | 0.85 | 0.91 | 0.49[0.4-0.58] | 0.46[0.36-0.59] | 13% | 6% |

**Abbreviations:** **AUC-** Area under the Receiver Operating Curve; **AUC^BS^**- .632+ bootstrap estimates of AUC; **AUC^*^**- apparent AUC; **CRC**- Colorectal Cancer; **95% CI**- 95 % Confidence Interval; **p_value**- apparent p-values without any adjustments; **p_value^adj^**- p-value after adjustment for multiple testing by Benjamini Hochberg method; **Se^BS^**- .632+ bootstrap estimates of Sensitivity; **Sp**- Specificity.
