# Supplementary material for: Multiplex screening of 275 plasma protein biomarkers to identify a signature for early detection of colorectal cancer
Source: Mol Oncol. 2019 Nov 13;14(1):8–21. doi: 10.1002/1878-0261.12591 (PMC6944100; doi:10.1002/1878-0261.12591)
Supplement: Supplementary file 4 — Table S3. The algorithms identified from the discovery set for (all/early/late) stage CRC detection. [file MOL2-14-8-s004.docx]

**Supplementary Table 3:** The algorithms identified from the discovery set for (all/early/late) stage CRC detection

| ***STAGE vs FREE OF NEOPLASM CONTROLS** | **ALGORITHMs** |
| --- | --- |
|  |  |
| **All stages CRC** | 4.5925+ (0.1844) **AREG**+ (0.0827) **CEA**+ (-0.0176) **GZMB**+ (-1.3093) **ITGAV**  + (0.1103) **KRT19**+ (-0.1961) **MCP1**+ (0.0697) **OPN**+ (-0.2746) **PON3**+ (0.2205) **TR** |
| **Early stages CRC** | -1.1987+ (0.5632) **AREG**+ (0.2179) **CEA**+ (-0.0619) **GZMB**+ (-1.0007) **ITGAV**  + (0.1119) **KRT19**+ (-0.0071) **MASP1**+ (-0.4285) **MCP1**+ (-0.4015) **PON3**  + (0.5095) **RARRES2**+ (-0.0732) **S100A4**+ (0.1816)**TR**+ (-0.0217) **TRAP** |
| **Late stages CRC** | 4.1340+ (0.0822) **AREG**+ (0.1727) **CEA**+ (0.0147) **IL6**+ (-0.0186) **ITGA11**  + (-2.2157) **ITGAV**+ (0.1866) **KRT19**+ (-0.1853) **MCP1**+ (0.0632) **NTproBNP**  + (0.2423) **OPN**+ (0.4183) **TR**+ (-0.1317) **TRAIL** |
| **AA** | -1.1987+ (0.5632) **AREG**+ (0.2179) **CEA**+ (-0.0619) **GZMB**+ (-1.0007) **ITGAV**  + (0.1119) **KRT19**+ (-0.0071) **MASP1**+ (-0.4285) **MCP1**+ (-0.4015) **PON3**  + (0.5095) **RARRES2**+ (-0.0732) **S100A4**+ (0.1816) **TR**+ (-0.0217) **TRAP** |

**Abbreviations:** **AA**- advanced Adenomas; **CRC**- colorectal Cancer

**All proteins abbreviations:** **AREG**- amphiregulin; **CEA**- carcinoembryonic antigen; **GZMB**- granzyme B; **IL6**- interleukin-6; **ITGA11**- integrin alpha 11; **ITGAV**- integrin alpha V; **KRT19**- keratin, type I cytoskeletal 19; **MASP1**- mannan-binding lectin serine protease 1 ; **MCP1**- monocyte chemotactic protein 1; **NTproBNP**- n terminal prohormone brain natriuretic peptide; **OPN**- osteopontin; **PON3**- paraoxonase 3; **RARRES2**- retinoic acid receptor responder protein 2; **S100A4**- protein S100-A4; **TR**- transferrin receptor protein 1; **TRAP**- tartrate-resistant acid phosphatase type 5; **TRAIL-** tnf related apoptosis inducing ligand.
