# Supplementary material for: Multiplex screening of 275 plasma protein biomarkers to identify a signature for early detection of colorectal cancer
Source: Mol Oncol. 2019 Nov 13;14(1):8–21. doi: 10.1002/1878-0261.12591 (PMC6944100; doi:10.1002/1878-0261.12591)
Supplement: Supplementary file 6 — Table S5. Diagnostic performance of the 9‐marker signature for detection of CRC in discovery and validation sets stratified by cancer location. [file MOL2-14-8-s006.docx]

**Supplementary Table 5:** Diagnostic performance of the 9-marker signature for detection of CRC in discovery and validation sets stratified by cancer location

| PROTEIN MARKERS DISCOVERED IN THE SIGNATURE | DISCOVERY SET | | VALIDATION SET  (As in screening population) |
| --- | --- | --- | --- |
|  | AUC^BS^ | AUC | AUC |
| AREG+ CEA+ GZMB+ ITGAV+ KRT19+ MCP1+ OPN+ PON3+ TR | **All CRC** | | |
|  | 0.92 | 0.92 | 0.76 |
|  | **Proximal Colon Cancer** | | |
|  | 0.94 | 0.98 | 0.70 |
|  | **Distal Colon Cancer** | | |
|  | 0.89 | 0.95 | 0.77 |
|  | **Rectal Cancer** | | |
|  | 0.90 | 0.95 | 0.78 |

**Abbreviations:** **AA**- advanced adenomas; **AUC-** area under the receiver operating curve; **AUC^BS^**- .632+ bootstrap estimates of AUC; **CRC**- colorectal cancer

**All proteins abbreviations:** **AREG**- amphiregulin; **CEA**- carcinoembryonic antigen; **GZMB**- granzyme B; **ITGAV**- integrin alpha V; **KRT19**- keratin, type I cytoskeletal 19; **MCP1**- monocyte chemotactic protein 1; **OPN**- osteopontin; **PON3**- paraoxonase 3; **TR**- transferrin receptor protein 1.
